# The Deubiquitinase USP47 Stabilizes MAPK by Counteracting the Function of the N-end Rule ligase POE/UBR4 in *Drosophila*

**DOI:** 10.1371/journal.pbio.1002539

**Published:** 2016-08-23

**Authors:** Dariel Ashton-Beaucage, Caroline Lemieux, Christian M. Udell, Malha Sahmi, Samuel Rochette, Marc Therrien

**Affiliations:** 1 Institute for Research in Immunology and Cancer, Laboratory of Intracellular Signaling, Université de Montréal, Montreal, Quebec, Canada; 2 Département de pathologie et de biologie cellulaire, Université de Montréal, Montreal, Quebec, Canada; Baylor College of Medicine, UNITED STATES

## Abstract

RAS-induced MAPK signaling is a central driver of the cell proliferation apparatus. Disruption of this pathway is widely observed in cancer and other pathologies. Consequently, considerable effort has been devoted to understanding the mechanistic aspects of RAS-MAPK signal transmission and regulation. While much information has been garnered on the steps leading up to the activation and inactivation of core pathway components, comparatively little is known on the mechanisms controlling their expression and turnover. We recently identified several factors that dictate *Drosophila* MAPK levels. Here, we describe the function of one of these, the deubiquitinase (DUB) USP47. We found that USP47 acts post-translationally to counteract a proteasome-mediated event that reduces MAPK half-life and thereby dampens signaling output. Using an RNAi-based genetic interaction screening strategy, we identified UBC6, POE/UBR4, and UFD4, respectively, as E2 and E3 enzymes that oppose USP47 activity. Further characterization of POE-associated factors uncovered KCMF1 as another key component modulating MAPK levels. Together, these results identify a novel protein degradation module that governs MAPK levels. Given the role of UBR4 as an N-recognin ubiquitin ligase, our findings suggest that RAS-MAPK signaling in *Drosophila* is controlled by the N-end rule pathway and that USP47 counteracts its activity.

## Abstract 2

In *Drosophila*, steady-state levels of the regulatory protein MAPK are found to be governed by the opposing actions of the deubiquitinase USP47 and components of the N-end rule pathway.

## Author Summary

The RAS-MAPK pathway plays a central role in the development of multicellular organisms, predominantly by regulating cell proliferation and differentiation. At its core, the pathway includes the RAS GTPase and three kinases (RAF, MEK, and MAPK) that transmit RAS signals through a phosphorylation cascade. Several factors have been discovered over the years that modulate signal transmission by altering the kinetics of phosphorylation/dephosphorylation of the core pathway components. In contrast, scant information is available on the mechanisms governing their expression and steady-state levels. Here, we report the characterization of a deubiquitinase (DUB) known as USP47, which stabilizes MAPK protein levels by opposing the activity of the proteasome, which is a major protein degradation machinery in eukaryotic cells. A search for enzymes that opposed USP47 activity identified several components of the N-end rule pathway centered on the poorly characterized POE/UBR4 E3 ligase. These components appear to work together to target MAPK for proteasome-mediated protein degradation and thereby reduce MAPK protein half-life. Together, this work identifies a new means by which RAS-MAPK signaling output is controlled in *Drosophila*. As the role of the N-end rule pathway remains largely uncharacterized in metazoans, this work opens new opportunities for studying its function in higher organisms.

## Introduction

The RAS-MAPK pathway is one of the principal signaling conduits controlling proliferation and differentiation in metazoans. Perturbations in pathway activity are closely associated to oncogenesis and developmental disorders [1,2]. Signaling through the small GTPase RAS is typically initiated by signal-receiving transmembrane receptors. Active RAS triggers the successive activation of the three pathway kinases: RAF, MEK, and MAPK/ERK. A complex network of factors is now known to regulate the steps leading up to MAPK activation [3,4]. To date, much effort has been devoted to identifying and characterizing the post-translational mechanisms that govern pathway signaling dynamics [3–5]. In the case of MAPK, MEK is the principal activator that operates by phosphorylating MAPK’s activation loop, and MAPK-specific phosphatases dephosphorylate these residues to inactivate the kinase. Other sources of regulation include scaffold proteins and factors that control subcellular localization [6]. As is the case for most other RAS-MAPK pathway components, very little is known about the processes that act to regulate MAPK abundance.

**Fig 1:**
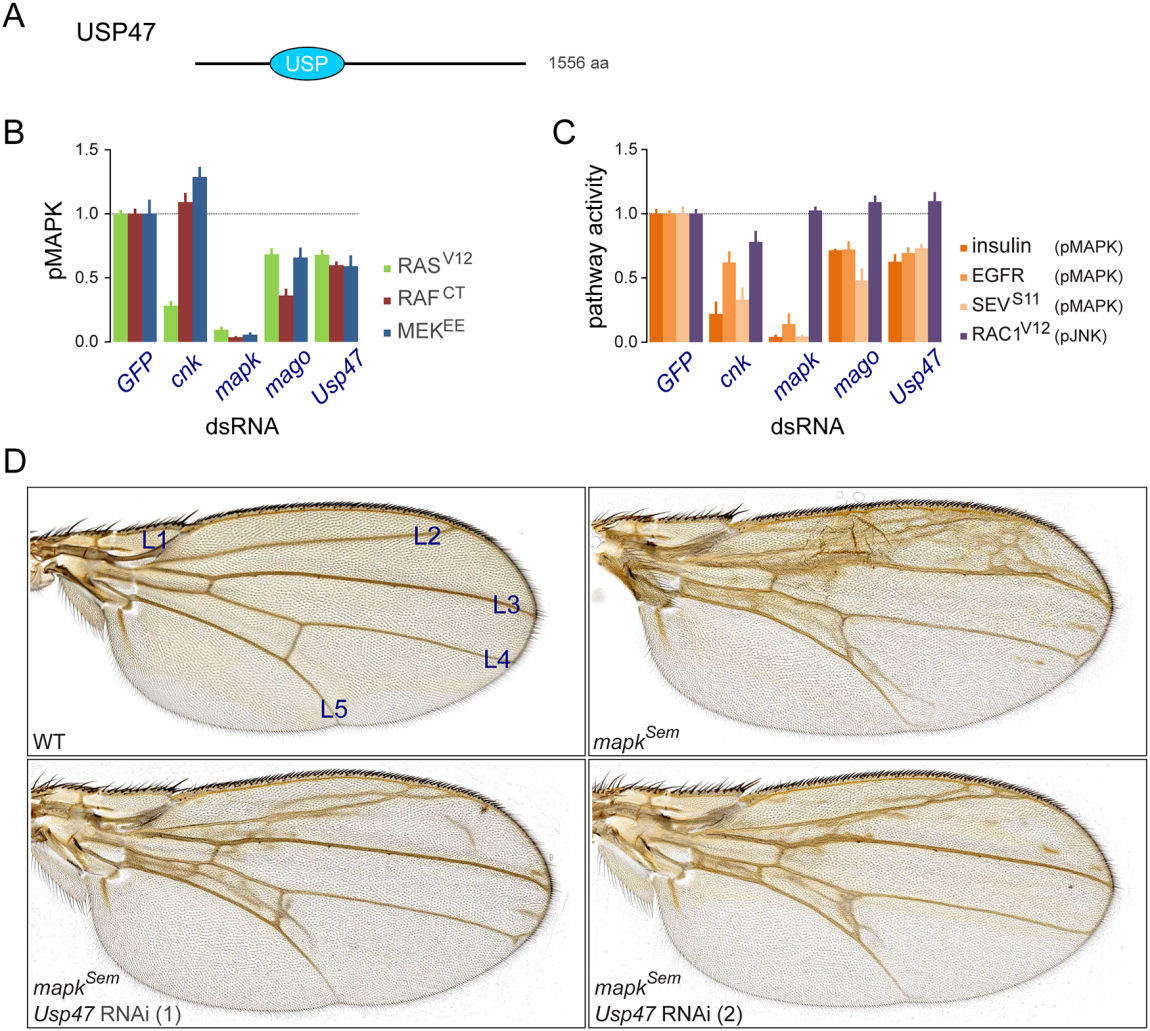
*Usp47* RNAi impacts RTK-RAS signaling downstream of MEK. (A) Schematic representation of the USP47 protein product with the position of the USP domain (ubiquitin specific protease catalytic domain) represented along with the amino acid length. (B) Epistasis analysis in *Drosophila* S2 cells employing constitutively active forms of RAS, RAF, and MEK to induce MAPK activation [8]. Phosphorylated MAPK was measured by quantitative microscopy and normalized to a *GFP* dsRNA control [7]. (C) MAPK activation is induced by three different receptor tyrosine kinases (RTKs) acting upstream: InR through insulin stimulation; EGFR-expressing cells stimulated with the Spitz ligand; or a heat-shock inducible constitutively activated form of Sevenless (Sev^S11^). JNK pathway activation induced by RAC1^V12^ is used as a negative control. (B-C) A dsRNA targeting the Exon Junction Complex (EJC) component *mago*, a factor also positioned downstream of MEK and known to reduce MAPK expression [7], is shown for comparison. The *cnk* dsRNA is a control for a factor known to act at the level of RAF [24]. (D) The *sal*^*EPv*^*-Gal4* drives expression in the wing pouch, which corresponds to a segment of the wing blade extending from the L2 wing vein to the L4-5 intervein with a weaker expression area extending in the periphery of this region [25,26]. A *mapk* gain of function *Sevenmaker* (*Sem*) mutant induced the production of extra wing vein material. Two different *Usp47* RNAi lines suppressing the extra wing vein material generated by *mapk*^*Sem*^ are shown (these correspond to the following fly lines: *Usp47* (1), VDRC line GD26027; *Usp47* (2), NIG line 5486R-3). Raw data for (B and C) can be found in S1 Data.

Recently, we reported the identification of a series of factors in a genome-wide RNAi screen that impacted RAS-MAPK signaling at a pre-translational level [7,8]. Unexpectedly, these factors were in large part associated with either the transcription or splicing of *mapk* pre-mRNA transcripts, indicating that this might be an important source of regulatory input into this pathway. Here, we focus on USP47 (also commonly referred to as UBP64E; FBgn0016756), a factor that was also identified in the aforementioned RNAi screen. USP47 is a deubiquitinase (DUB) of the ubiquitin specific protease (USP) family (Fig 1A) [9]. It has previously been associated with the regulation of the transcription factors TTK (*tramtrack*; FBgn0003870) and SLBO (FBgn0005638) in *Drosophila* [10,11]. Its human orthologue, USP47 interacts with the beta-TRCP E3 ligase complex [12] and has been found to regulate base-excision repair (BER) by controlling the levels of Polymerase β [13,14]. USP47 has also been linked to axonal growth by working antagonistically to the E3 ligase CHIP in regulating the microtubule severing protein katanin-p60 [15]. Additionally, it has been associated with cell—cell adhesion through its ability to prevent E-cadherin degradation, which stabilizes adherens junction formation [16]. Finally, USP47 has recently been shown to control Wnt signaling by controlling β-catenin stability both in human and *Drosophila* cells [17].

In the present study, we show that USP47 acts post-translationally to stabilize *Drosophila* MAPK (*rl*; FBgn0003256) levels both in cell culture and in vivo, and that this mechanism is independent of MAPK activity. Moreover, this event appears to be specific to MAPK, as no other core components of the pathway nor other *Drosophila* MAPK-like proteins, JNK (*bsk*; FBgn0000229) and p38B (FBgn0015765), are modulated by USP47 function. To identify putative MAPK destabilizing components that might act in opposition to USP47, we conducted a targeted RNAi screen for genetic interactors of *Usp47* depletion. Interestingly, this approach led to the identification of three factors, namely, the E2 ubiquitin conjugating enzyme UBC6 (FBgn0004436) and the putative E3 ligases POE (FBgn0011230; the fly UBR4 ortholog) and UFD4 (FBgn0032208), which restored MAPK levels when individually co-depleted with USP47. Moreover, in a search for additional factors that act similarly to the newly identified E2/E3 enzymes, we found that KCMF1 (FBgn0037655), a zinc finger-containing protein that physically interacts with POE, also antagonized USP47 activity.

Remarkably, UBC6, UBR4, and UFD4 are all linked to a mechanism called the N-end rule pathway, which has mostly been studied in yeast and whose relevance to metazoan cells remains largely unknown. The N-end rule pathway is a ubiquitin/proteasome-dependent protein degradation process that hinges on the recognition of a degron located at the N-terminal extremity (N-degron) of peptides and whose principal determinant is the identity of the N-terminal amino acid. This N-terminal amino acid is recognized by the ubiquitin protein ligase E3 component n-recognin (UBR) box domain of a class of E3 ligases called N-recognins, which includes POE/UBR4 [18–21]. UBC6 is the fly ortholog of RAD6, which is the main E2 enzyme that functions with UBR box N-recognins [18,22], whereas UFD4 is a poorly characterized HECT domain E3 ligase whose yeast homolog, Ufd4, has also been linked to N-end rule function [23].

These new findings suggest that the N-end rule pathway plays a role in establishing MAPK levels and, together with previously characterized pre-translational mechanisms, fine-tunes MAPK signaling output during development.

## Results

### USP47 Modulates RAS-MAPK Signaling Downstream of MEK

We initially identified *Usp47* in a genome-wide RNAi screen for factors modulating RAS^V12^-induced (*Ras85D*; FBgn0003205) pMAPK activity in *Drosophila* S2 cells [8]. Like most of the factors identified in this screen, *Usp47* was found to act downstream of MEK (*Dsor1*; FBgn0010269). Indeed, signaling by constitutively active RAS, RAF, or MEK were all equally suppressed by a dsRNA targeting *Usp47* to a degree that was similar to that induced by *mago* (FBgn0002736), a factor known to act downstream of MEK (Fig 1B) [7]. In contrast, *cnk* (FBgn0021818), a well characterized regulator of RAF activation [24], reduced pMAPK signaling in the active RAS assay, but had no impact in active RAF (FBgn0003079) or MEK assays (Fig 1B). *Usp47* also demonstrated a broad capacity to modulate MAPK signaling in different contexts; signal induced by the Insulin-like Receptor (*InR*; FBgn0283499), Sevenless (*sev*; FBgn0003366), or Epidermal Growth Factor Receptor (*Egfr*; FBgn0003731) was suppressed to the same extent by *Usp47* RNAi (Fig 1C). JNK activity induced by RAC1^V12^ (*Rac1*; FBgn0010333) was, however, not modified by *Usp47* depletion (Fig 1C).

We next conducted genetic interaction experiments to validate *Usp47*’s newfound function in vivo. In flies, RAS-MAPK signaling is important in many aspects of development, including the induction of photoreceptor and cone cell differentiation in the eye [27] as well as in promoting the formation of veins during wing development [28,29]. Overexpression of a *mapk* gain-of-function allele (*mapk*^*Sem*^) [30] in *Drosophila* wings using a *sal*^*EPv*^*-Gal4* driver led to the production of extra wing vein material (Fig 1D) [31]. The severity of this ectopic wing vein phenotype was significantly reduced by co-expressing *Usp47* RNAi (Fig 1D). In the eye, genetic lesions that lower pathway activity cause a readily observable rough eye phenotype in adults that is due to missing photoreceptor cells (S1A Fig) [32,33]. Two alleles of *Usp47* (*Usp47*^*Δ1*^ and *Usp47*^*Δ2*^) that reduce USP47 protein expression [10] were found to increase the severity of the rough eye phenotype of *rl*^*1*^ homozygous flies carrying a hypomorphic allele of *mapk* (S1A Fig). Lastly, similar observations were also made in hemizygotes for *csw*^*lf*^ (S1B Fig), a dominant negative form of the *csw/shp-2* (FBgn0000382) phosphatase that regulates RAS activity [34]. In sum, these experiments support our cell culture-based observations pointing towards a positive regulatory role for USP47 in MAPK signaling.

### USP47 Regulates MAPK Levels Post-translationally

**Fig 2:**
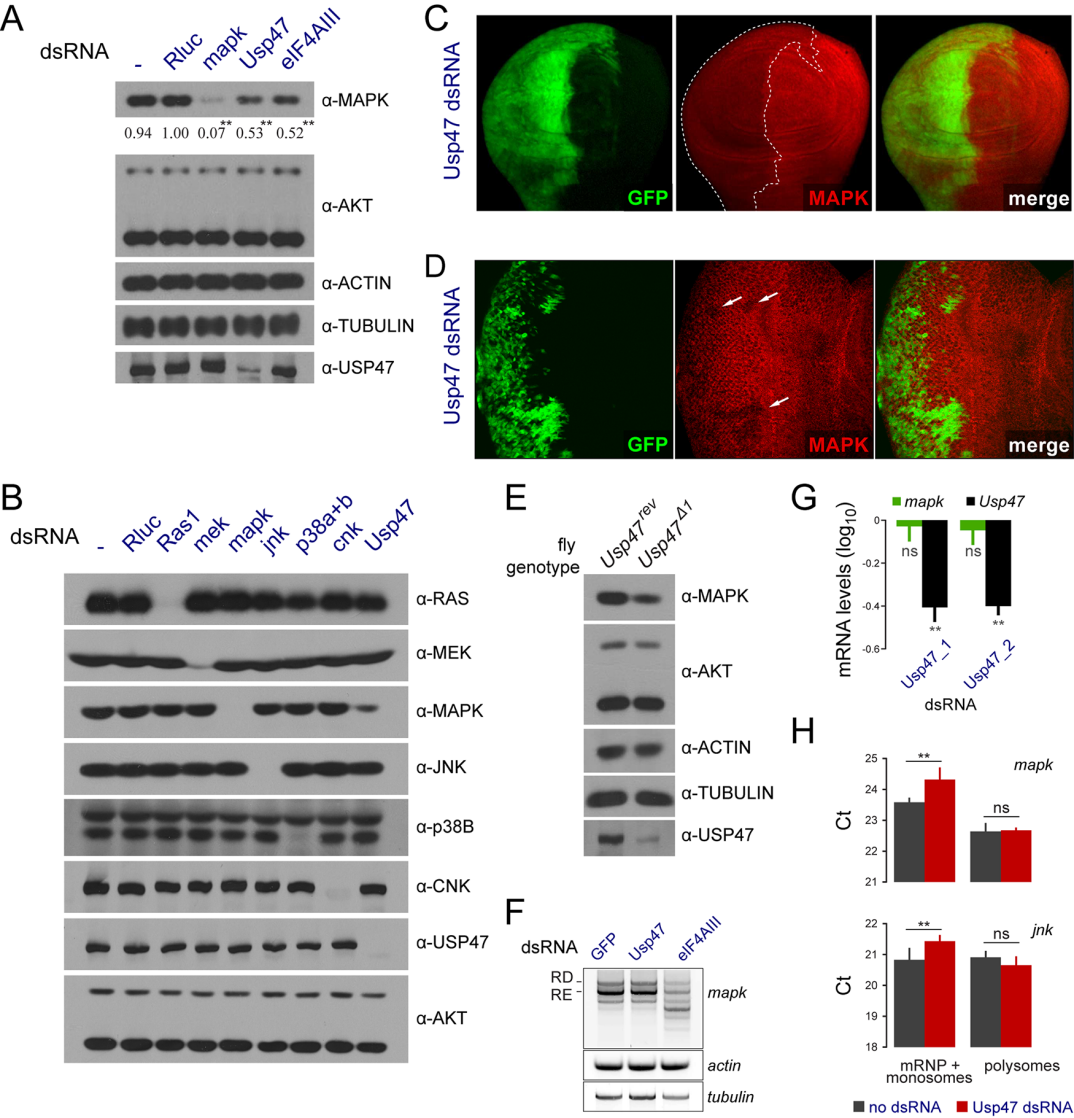
*Usp47* regulates MAPK levels. (A) *Usp47* depletion causes a reduction in MAPK levels probed by immunoblot. S2 cells were treated with the indicated dsRNAs. The EJC component *eIF4AIII* was used as a control for a factor that reduces MAPK expression [7]. ACT5C (FBgn0000042), α-TUBULIN (FBgn0003884), and AKT (FBgn0010379) levels (loading controls) were not impacted by *Usp47* depletion. Densitometry quantifications are provided for MAPK levels normalized to the AKT loading controls (similar quantification results are obtained by using either the ACTIN or the TUBULIN controls for normalization (see S1 Data). Two AKT isoforms (short and long) are detected in S2 cells by western blot. We exclusively used the shorter variant as loading control for quantification purpose, as its levels are in the same detection range as MAPK from the same sample lysate. The levels of the long AKT isoform were slightly variable across experiments and thus were not used for quantification. Their experimental variation was likely caused by their lower abundance, which in turn would produce signals outside the linear range of the film. This experiment was performed in seven replicates, and *p*-values were calculated (paired two tailed Student’s *t* test) comparing values to the *Rluc* control (*: *p* < 0.05; **: *p* < 0.01). (B) *Usp47* depletion did not alter the levels of other RAS-MAPK pathway components (RAS, MEK, and CNK), nor of two other MAPK family kinases (JNK and p38). As AKT, ACTIN, and TUBULIN were found to be interchangeable loading controls in experiments that address the impact of USP47 on MAPK levels (A), we decided for convenience to monitor only AKT levels as loading control here and in most subsequent experiments. AKT was selected over ACTIN or TUBULIN because its transcript levels, according to modENCODE, are similar to those of mapk (unlike for actin or tubulin, which are 200–500-fold more highly expressed), and thus allowed the comparison of similarly expressed genes. Importantly, AKT levels are not known to be modulated by MAPK signaling. For instance, we never observed variations in AKT levels following perturbations in RAS-MAPK signaling [7,8], which is also reflected here in (B). Finally, the other monitored proteins in (B) also serve as points of comparison to infer that AKT levels are not impacted by Usp47 depletion. (C-D) MAPK levels are also reduced by *Usp47* knockdown in vivo. Tissue expressing *Usp47* RNAi (using the VDRC RNAi line: GD26027) is marked with GFP. (C) The expression of *Usp47* RNAi was induced using an *engrailed-Gal4* promoter that drives expression in the posterior segment of wing discs. (D) Heat shock-induced flip-out clones expressing *Usp47* RNAi in eye imaginal discs also show reduced MAPK levels. (E) MAPK protein levels are reduced in flies bearing the *Usp47*^*Δ1*^ mutation. Lysates were prepared from adult flies homozygous for either the hypomorphic *Usp47*^*Δ1*^ allele or a *Usp47*^*rev*^ control (a WT *Usp47* line generated by a precise excision of the P element used to generate the *Usp47*^*Δ1*^ allele). (F-G) *Usp47* depletion does not impact the *mapk* transcript levels. (F) *mapk* transcript levels were measured following dsRNA treatment using an RT-PCR assay targeting the 5′ and 3′ UTRs that amplifies the RD and RE splice isoforms of *mapk*. *eIF4AIII* (FBgn0037573) dsRNA is used as a control that alters *mapk* splicing. *Act5C* and *γ-Tubulin* (*γTub23C*; FBgn0260639) loading controls are also shown. (G) *mapk* transcript levels measured by qPCR (normalized to *GFP* dsRNA control) are not impacted by two different dsRNA reagents targeting *Usp47* (*p* > 0.05) compared to an untreated control, while *Usp47* levels are decreased in both cases (**: *p* < 0.01). “ns” denotes not significant. (H) Polysomal loading of *mapk* transcripts is not altered by *Usp47* depletion. The polysome and messenger ribonucleoprotein (mRNP)/monosome fractions were separated on a sucrose gradient. qPCR was used to assay *mapk* and *jnk* mRNA transcript levels in both fractions. A slight decrease of *mapk* transcript abundance (**: *p* < 0.01) was observed in the mRNP/monosome fractions upon *Usp47* depletion. However, this was observed for *jnk* transcripts and was not accompanied by a concomitant variation in the polysome fraction. “ns” denotes not significant. Raw data for (A, G, and H) can be found in S1 Data.

Like many of the candidates identified in our initial genome-wide screen, *Usp47* RNAi was found to cause a decrease in MAPK protein levels (Fig 2A) [8], whereas it did not visibly alter the expression levels of JNK (FBgn0000229) and p38b (FBgn0024846), two other members of the MAPK family, nor of other RAS-MAPK pathway proteins (Fig 2B). This effect was also observed in vivo, where knocking down *Usp47* in both fly larval wing and eye imaginal discs caused a reduction in MAPK protein levels (Fig 2C and 2D). Moreover, lysates prepared from *Usp47*^*Δ1*^ homozygotes were found to contain less MAPK protein than WT controls (Fig 2E). A clear distinction between *Usp47* and other candidates from our initial screen arose when examining their impact on *mapk* mRNA. Indeed, while the other factors caused changes in *mapk* mRNA levels or splicing, *Usp47* had no impact at these levels (Fig 2F and 2G). Moreover, it did not cause a change in the rate of *mapk* translation as measured by polysomal loading of *mapk* mRNA transcripts (Fig 2H). Thus, regulation of MAPK levels by USP47 does not appear to be pre-translational.

**Fig 3:**
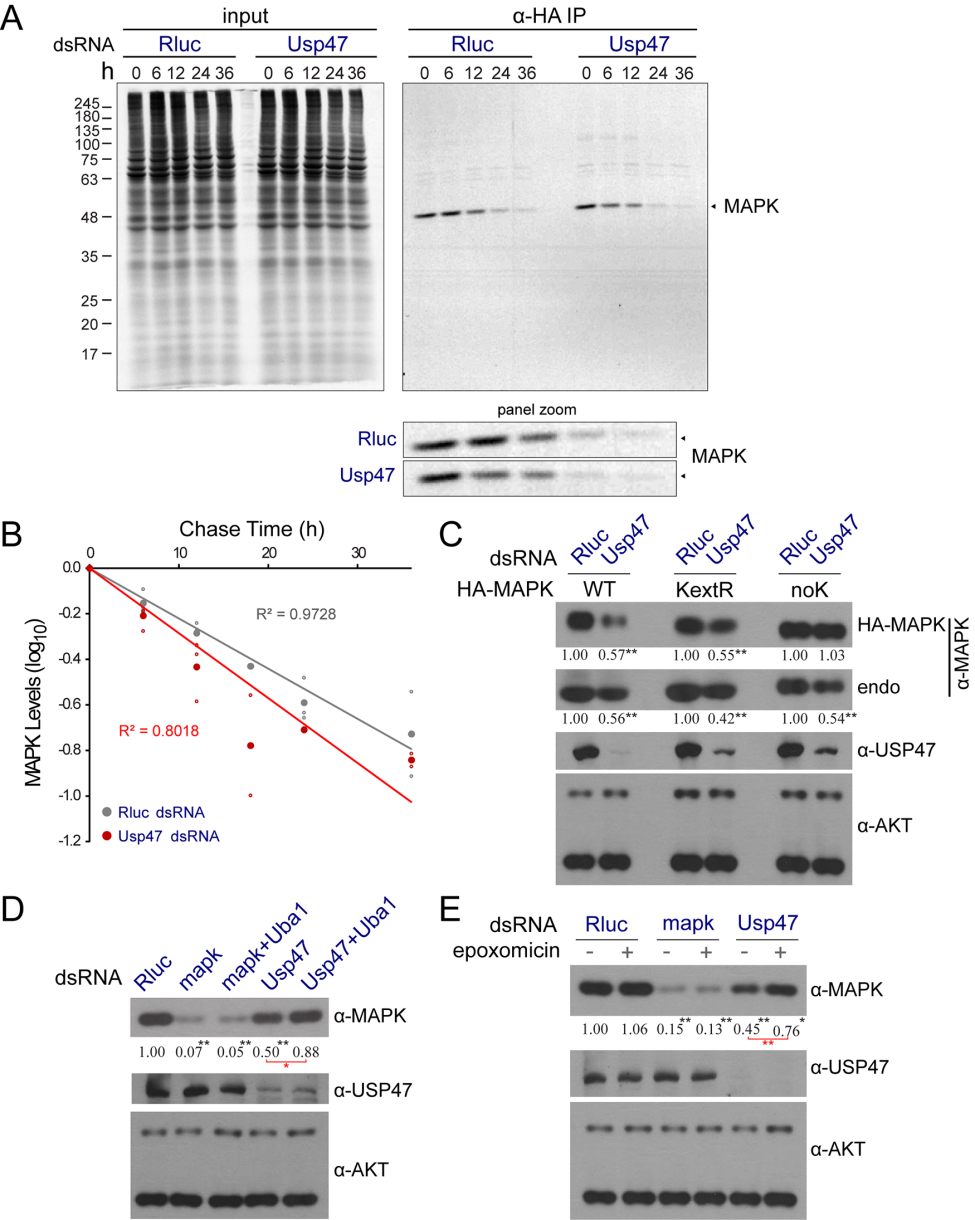
USP47 acts post-translationally on MAPK. (A) Pulse-chase experiments of [35S]-methionine labeled MAPK show an impact on MAPK half-life. S2 cells were treated with the indicated dsRNAs for 5 d followed by 6 h of [35S]-methionine labeling. Cell lysates were then prepared at the indicated time following the labeling period. (B) Densitometry quantification of three separate replicates of the experiment presented in A. MAPK levels were normalized to that of three bands from the input gel. Based on these results, MAPK half-life was reduced from 13.68 h (*Rluc* dsRNA controls) to 10.34 h (*Usp47* dsRNA). (C) *Usp47* dsRNA was added to cells stably expressing wild-type exogenous HA-tagged MAPK, causing a reduction in MAPK levels. Similar cell lines were established in which the 10 predicted surface exposed lysines (MAPK^KextR^; external lysines were selected based on the structure of ERK2 and correspond to residues 67, 68, 92, 127, 151, 164, 177, 216, 220, 283, and 313 of *Drosophila* MAPK) or all lysines (MAPK^noK^) were switched to arginines. Only mutation of all of the lysines abrogated the sensitivity of MAPK levels to *Usp47* depletion. (D) Depletion of the *Drosophila* E1 ligase, *Uba1* (FBgn0023143; see S5 Table for qPCR validation of RNAi reagent), rescues the impact of *Usp47* RNAi on MAPK levels. (E) Treating S2 cells with the epoxomicin proteasome inhibitor restored MAPK levels in a *Usp47* depleted context. (C–E) Densitometry quantifications are provided for MAPK levels (normalized to the AKT loading controls). All immunoblots were performed in triplicate or more, and *p*-values were calculated (paired two tailed Student’s *t* test) comparing values to the *Rluc* control (*: *p* < 0.05; **: *p* < 0.01). *t* tests performed on other samples are shown in red. Raw data for (B–E) can be found in S1 Data.

In order to determine if USP47 is acting at a post-translational level, we resorted to pulse-chase metabolic labeling with [35S]-methionine. We found labeled MAPK to be degraded more rapidly in cells treated with *Usp47* RNAi (Fig 3A and 3B). Indeed, in comparison to a half-life of 13.68 h in control conditions, the half-life of MAPK went down to 10.34 h upon USP47 depletion, which is consistent with the ~50% reduction of steady-state levels of MAPK typically seen by western blot following 4 d of dsRNA treatment (Fig 2A). Importantly, an exogenous HA-MAPK cDNA, but not HA-JNK or HA-p38B, stably expressed in S2 cells also displayed sensitivity to *Usp47* dsRNA (S2A Fig). Taken together, these experiments strongly suggest that USP47 functions post-translationally to specifically regulate MAPK protein levels.

One likely mechanism for post-translational control of MAPK levels by a deubiquitinase would be the removal of ubiquitin moieties from specific lysine residues on MAPK. As no ubiquitin ligases are associated to the regulation of MAPK expression in *Drosophila*, and as we had not identified any such factors in our genome-wide RNAi screen, we next sought to determine if the ubiquitin proteasome system (UPS) was required for the destabilization of MAPK following *Usp47* knockdown. We found that co-depletion of *Uba1*—the sole ubiquitin-activating enzyme (E1) in *Drosophila*—and *Usp47* by RNAi rescued MAPK levels to near baseline (Fig 3D). Proteasome inhibition using epoxomicin had a similar effect (Fig 3E). Thus, these results indicate that the ubiquitin-proteasome system acts in opposition to USP47 to control MAPK protein levels.

Despite these indicators of UPS involvement, we did not, however, detect any polyubiquitination of MAPK when co-expressing HA-tagged ubiquitin in S2 cells (S3 Fig), which contrasted with the readily observable ubiquitination of controls such as RAF and KSR (FBgn0015402) (S3 Fig). Since ubiquitin might likely be attached to an exposed lysine at the surface of MAPK, we also mutated all predicted surface-exposed lysine residues. Unexpectedly, MAPK modified in this manner still responded to *Usp47* RNAi to a degree comparable to WT (Fig 3C). In contrast, mutation of all lysine residues did abrogate the effect of *Usp47* (Fig 3C). However, this latter mutant was not phosphorylatable by MEK (unlike the WT or surface-exposed lysine mutant; S2B Fig), thus suggesting that its structure is significantly altered. Accordingly, mutations predicted to disrupt the N- or C-lobe of the kinase domain of MAPK also displayed a reduced sensitivity to *Usp47* RNAi (S2C Fig). Moreover, deletion of MAPK’s C-terminal α_L16_ helix, which interacts with the N-lobe and stabilizes it [6,35,36], also led to a loss of sensitivity to *Usp47* knockdown (S2D Fig). Thus, while we could not identify specific MAPK lysine residues as potential ubiquitination sites to explain the requirement in USP47 activity, our data strongly suggest that a properly folded MAPK protein is required. This conclusion implies that turnover of misfolded or unstructured MAPK protein products is unlikely to account for the observed impact of USP47. Still, it is possible that MAPK is ubiquitinated on one of its buried lysines. However, USP47’s post-translational access to buried lysines would have to occur in a manner that does not involve a prior significant disruption of MAPK structure.

One alternative way in which buried lysines might be readily targeted for ubiquitination is during co-translational quality control that occurs when the nascent peptide is not yet fully folded [37]. To investigate this possibility, we again used [35S]-methionine metabolic labeling, but, unlike the previously described pulse-chase experiment (Fig 3A), labeling was performed for a shorter period (4 h), after which MAPK levels were immediately assayed. The newly synthesized MAPK proteins marked in this manner did not show any changes when we compared *Usp47* RNAi treated samples to negative controls (S4 Fig). Therefore, this indicates that USP47 is not acting co-translationally on newly synthesized MAPK peptides during a hypothetical ribosomal quality control step.

We next sought to examine the importance of two key regions of MAPK that are linked to its function and regulation. First, we mutated the tyrosine and threonine residues of the activation segment of MAPK to see if phosphorylation by MEK, which is required for MAPK activation, could have an impact on regulation by USP47. However, we found that such a TEY→AEF MAPK mutant was still sensitive to *Usp47* depletion (S2E Fig). We then mutated MAPK’s D-site recruitment site (also known as “DRS”) that binds to MAPK D-site docking domains, which are present on some MAPK substrates and regulators [6]. The DRS mutant still responded to *Usp47* depletion (S2F Fig). This is also in line with our in vivo data showing that *mapk*^*Sem*^, a MAPK gain-of-function point mutation that disrupts the DRS [30], is also sensitive to *Usp47* knockdown (Fig 1D). In addition to the DRS, MAPK comprises an F-site recruitment site (FRS) that is involved in binding to substrates such as Elk1 and c-Fos, and which is exposed only following MAPK activation [6]. Since the non-activatable AEF mutant still responds to USP47 depletion, this implies that the FRS also does not play a role in determining sensitivity to USP47. Finally, in parallel to this, we tested the impact of *Usp47* RNAi on mammalian ERK1 and ERK2 stably expressed in S2 cells. Interestingly, we found them to respond to the same extent as *Drosophila* MAPK (S2G Fig). Together, these results indicate that post-translational regulation of MAPK activity by dynamic phosphorylation is likely uncoupled from regulation by USP47. Also, because the evolutionary distant mammalian ERK1/2 also respond to *Usp47* RNAi, sensitivity to USP47 is most likely determined by an evolutionarily conserved structural feature of MAPK that is distinct from the activation segment, DRS, or FRS.

### Identification of Factors that Antagonize USP47

**Fig 4:**
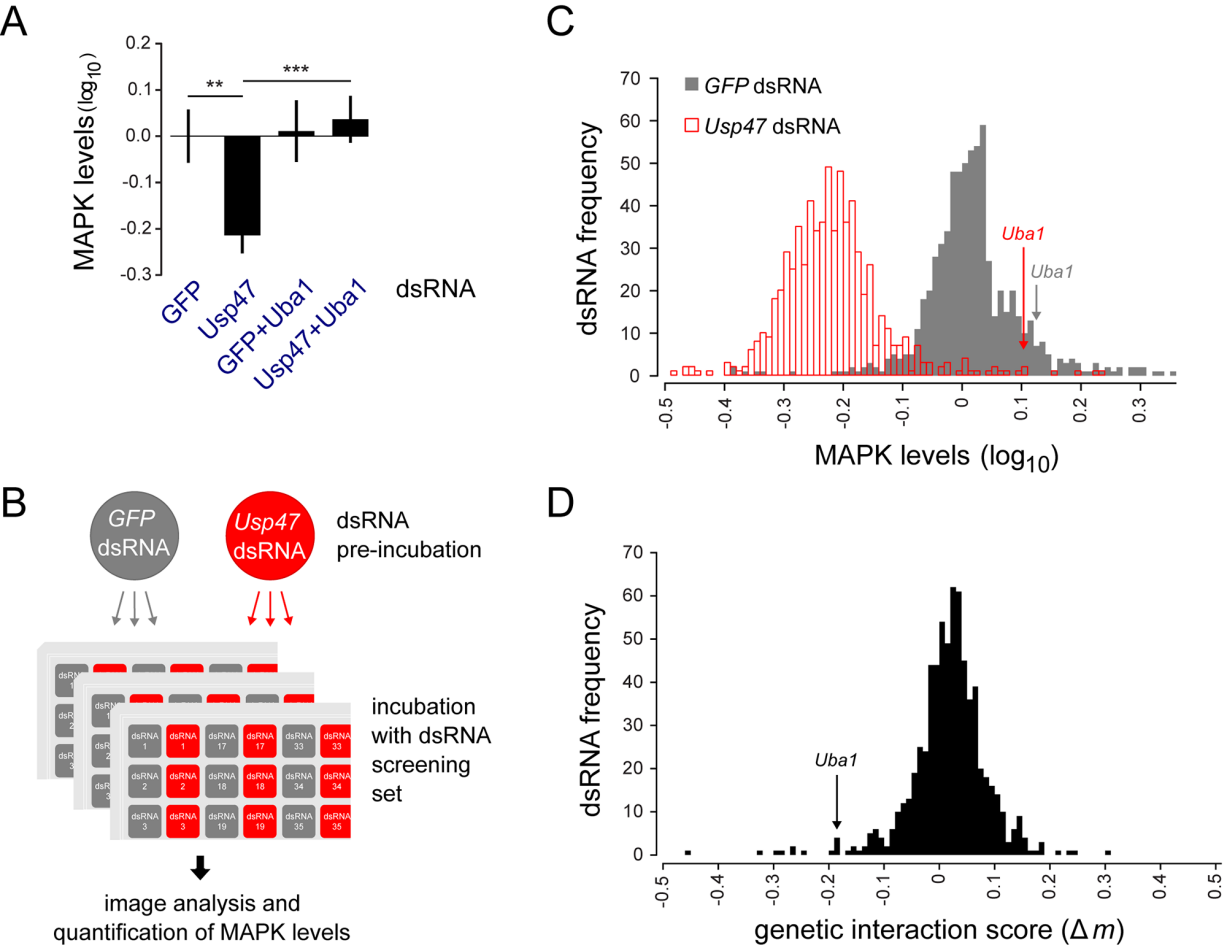
An RNAi screen to identify factors that modify the impact of USP47 on MAPK levels. (A) The rescue effect of *Uba1* depletion can be robustly measured in a plate-based quantitative microscopy assay suitable for large-scale screening. *Usp47* and *Uba1* co-depletion significantly restored MAPK levels (***: *p* < 0.001) compared to a *Usp47* single depletion (**: *p* < 0.01). *Uba1* single depletion did not significantly alter MAPK levels compared to *GFP* dsRNA treated control cells (used for normalization). In this experiment, S2 cells were pre-incubated with either *GFP* dsRNA (first and third sample) or with *Usp47* dsRNA (second and fourth sample) for 3 d. The cells were then distributed in 384 well plates containing the indicated dsRNAs and incubated for another 3 d. Following dsRNA treatment, anti-MAPK stained cells were imaged and analyzed by high-content microscopy. (B) Screening strategy for a large-scale RNAi screen focused on ubiquitin-proteasome associated factors. RNAi treatment and MAPK quantification for the ubiquitin-proteasome dsRNA set was performed as in A. Each condition was tested in quadruplicate. (C) Histogram showing the distribution of results from the *Usp47* RNAi screen. The frequency (number of dsRNAs) is displayed on the *y*-axis. MAPK levels (*x*-axis) were normalized to GFP dsRNA treated cells. The *Usp47* co-depleted cells show a clear shift towards a reduction in MAPK levels. The *Uba1* dsRNA control stands out from the lot, as it completely counteracts the *Usp47* dsRNA. (D) Distribution of *Usp47* genetic interaction scores (Δ*m*; *x*-axis) for the candidate dsRNAs (frequency displayed on *y*-axis) tested in the *Usp47* RNAi screen. The negative Δ*m* obtained for Uba1 is consistent with the observed alleviation of the MAPK level reduction. Raw data for (A) can be found in S1 Data. Numerical data for (C and D) can be found in S1 Table.

Given that proteasome inhibition and *Uba1* knockdown counteracted *Usp47* depletion by restoring MAPK levels, we reasoned that at least one E2 ubiquitin conjugating enzyme and E3 ligase might also have similar properties. Previous studies have shown that RNAi co-depletion can be used as an effective means to identify related factors and establish epistasis relationships [38–40]. Thus, in order to identify such specific regulators involved in counteracting USP47 activity, we sought to isolate candidates that, like *Uba1*, interacted genetically with *Usp47*. Genetic interaction is defined here as a dual depletion effect that differs significantly from the sum of the individual depletion effects (S5 Fig) [39,41,42]. We used an immunofluorescence assay to quantify MAPK level variations that allowed us to obtain both a robust reduction following *Usp47* RNAi as well as a rescue effect induced by co-depletion of *Uba1* (Fig 4A). We employed this assay to conduct a targeted RNAi screen using a focused dsRNA library encompassing factors linked to ubiquitin-proteasome function (S1 Table; S1 Text) that might interact genetically with *Usp47*. RNAi reagents were tested in combination with a *GFP* control dsRNA or in co-depletion with *Usp47* dsRNA (Fig 4B and 4C, S6A Fig and S1 Table).

In order to distinguish specific modifiers of *Usp47* from other candidates that might simply alter MAPK levels in an unrelated process (such as transcription or splicing regulation), we derived a genetic interaction score (Δ*m*) that was used for primary hit selection (S5A Fig and S1 Text). The Δ*m* indicates the difference between the expected and observed outcomes of the combined depletion of *Usp47* and gene *x*. Absence of genetic interaction occurs when the expected and observed outcomes are equivalent (Δ*m* = 0), such as in the case of a purely additive effect when combining two dsRNAs. An aggravating genetic interaction is said to occur when the observed effect of combined *Usp47* and *x* depletion is greater than the expected sum of the individual depletion effects (Δ*m* > 0; *x* is an enhancer of *Usp47*). Conversely, an alleviating interaction (Δ*m* < 0; suppressor) occurs when the observed depletion effect of *Usp47* and *x* is lesser than the expected sum (S5B and S5C Fig). Using *Uba1* as a reference, we selected a Δ*m* cutoff to guide our choice of primary hits for follow-up validation (Fig 4D, S6B and S6E Fig and S1 Text). A number of components from the basal UPS displayed genetic interaction with *Usp47*, and, as might be expected, many of these factors also caused a reduction in cell count indicative of an impact on cell viability (S1 Table). We thus used a more stringent cutoff for factors that caused an appreciable reduction in cell number (S6E Fig and S1 Text), thus removing some UPS factors from our primary hit selection in order to focus on non-cell lethal candidates.

A total of 55 primary hits were recovered from the screen and further selected for validation using independent dsRNA reagents (S2 Table). In our validation experiments, only candidates alleviating the impact of *Usp47* dsRNA on MAPK levels were confirmed (Δ*m* < 0) (S2 Table). The absence of aggravating genetic interactions (Δ*m* > 0) could signify that this type of regulation is not operating on USP47/MAPK. However, as *Usp47* RNAi only partially depletes MAPK levels compared to the effect of *mapk* dsRNA itself (Fig 2A), it might be expected that another DUB is acting redundantly to protect MAPK from complete degradation, in which case this factor should display an aggravating genetic interaction with *Usp47* RNAi (S5 Fig). In order to further explore this hypothesis—and the possibility that a redundant DUB might have been missed in our primary screen—we re-screened the other 41 predicted DUBs in the fly genome using a set of distinct dsRNAs. This second experiment confirmed our primary screen data, yielding no other DUBs that significantly impacted MAPK levels, either alone or when co-depleted with *Usp47* (S7 Fig and S3 Table). Taken together, these results suggest that *Usp47* is not acting redundantly with another DUB and that the partial impact on MAPK levels must have another cause. For instance, dsRNA may not allow for knockdown below a certain threshold, and the degradation machinery may not be able to access the entire pool of MAPK or de novo synthesis of MAPK compensates for *Usp47* RNAi-induced destabilization.

**Fig 5:**
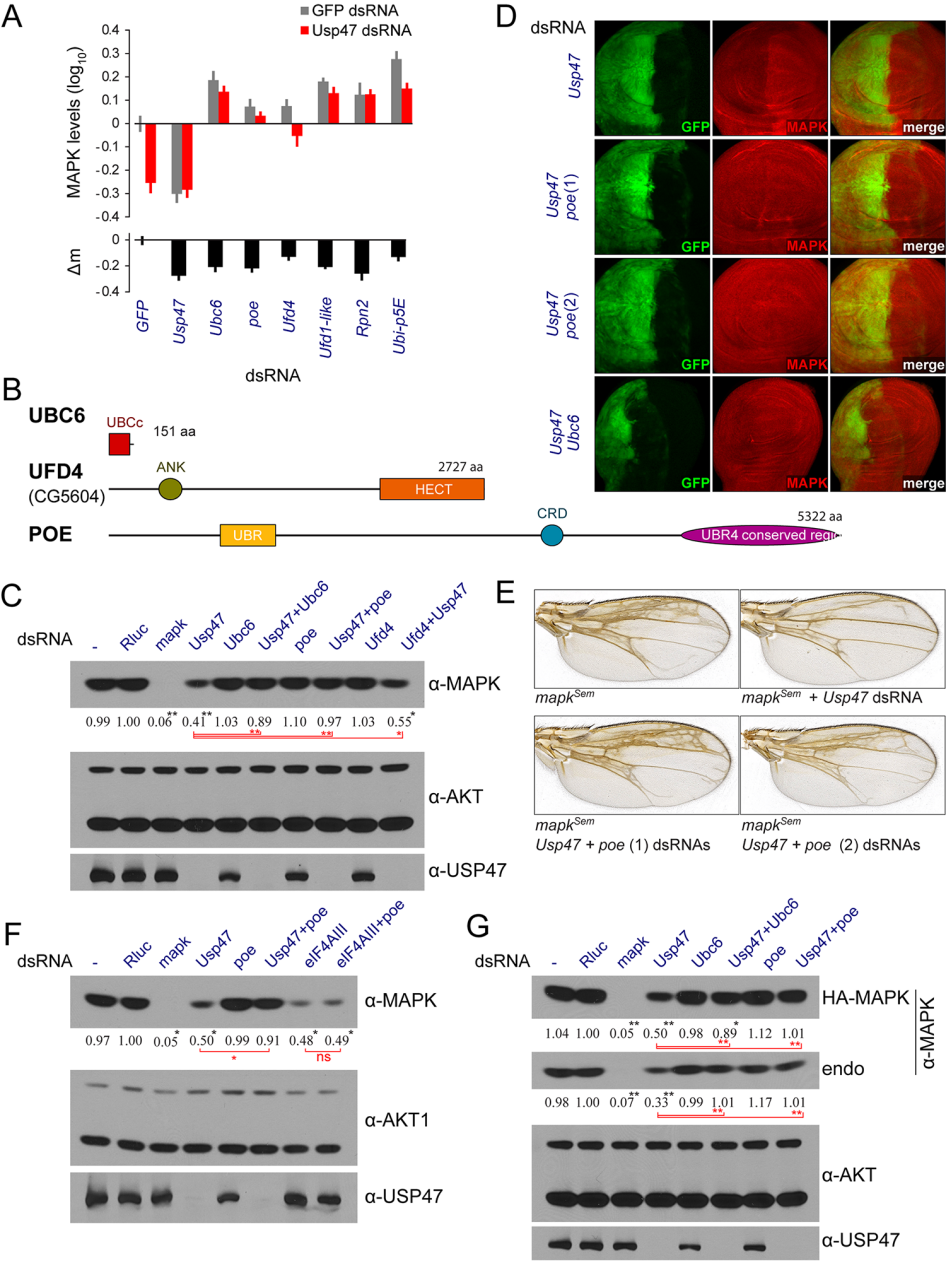
*Ubc6*, *poe*, and *Ufd4* dsRNAs alleviate *Usp47*’s impact on MAPK levels. (A) Selected hits from the *Usp47* co-depletion RNAi screen (qPCR validation in S5 Table). After validation with independent dsRNA reagents, our set of candidates included one E2 conjugating enzyme, *Ubc6*, and two E3 ligases, *poe* and *Ufd4*. The set also included proteasome components such as *Rpn2* (FBgn0028692) and ubiquitin genes such as *Ubi-p5E* (FBgn0086558). *Ufd1-like*, a proteasome-associated factor also linked to endoplasmic-reticulum-associated protein degradation (ERAD) was also present in our set (see S2 Table for full validation screen data). Data shown here is from the RNAi validation experiment. All MAPK levels are normalized to *GFP* dsRNA controls. The numerical data presented in this panel can be found in S2 Table. (B) Schematic representations of the UBC6, UFD4, and POE protein products drawn to scale with the position of identifiable domains and regions (UBCc: ubiquitin-conjugating enzyme E2 catalytic domain; ANK: ankyrin repeat motif; HECT: E3 ligase domain [HECT stands for “homologous to the E6-AP carboxyl terminus”]; UBR: ubiquitin protein ligase E3 component n-recognin domain [also known as UBR box motif]; CRD: cysteine rich domain). Amino acid lengths are also shown. (C) Western blot experiments confirm that *Ubc6*, *poe* and *Ufd4* depletion rescue endogenous MAPK levels in *Usp47* depleted cells. The rescue mediated by *Ufd4* depletion is weaker, possibly due to comparatively moderate depletion efficiency (see S5 Table). (D) Co-depletion experiments conducted in third instar *Drosophila* wing discs show that *poe* and *Ubc6* can rescue *Usp47* depletion in vivo. The *engrailed-gal4* driver was used to drive expression of RNAi in the posterior segment (GFP-positive) of the disc. *Ubc6* RNAi expression caused extensive larval lethality. Those wing discs that could be recovered were of reduced size (cell lethality was also problematic in confirming *Ubc6* depletion by qPCR (see S5 Table). The RNAi lines used in this experiment correspond to the following VDRC lines: *Usp47*, GD26027; *poe* (1), KK108296; *poe* (2), GD17648; *Ubc6*, GD23229. The depletion of the *Usp47*, *poe*, and *Ubc6* transcripts were also measured by qPCR (see S5 Table). (E) The extra wing vein material induced by *mapk*^*Sem*^ expression under the control of *sal*^*EPv*^-*Gal4* is suppressed by *Usp47* RNAi (see Fig 1D). Co-expression of *poe* dsRNA counteracts *Usp47* RNAi and restores the extra wing vein phenotype. The RNAi lines used here are the same as in C. (F) Pre-translational downregulation of mapk expression is not rescued by *poe* RNAi. Knocking down the EJC component *eIF4AIII* reduces MAPK levels due to altered splicing of the *mapk* transcript. This, unlike the depletion produced by *Usp47* dsRNA, is not rescued by co-depleting POE. (G) Exogenous MAPK levels measured by immunoblot are rescued by co-depletion of *Usp47* with *poe* or *Ubc6*. An *HA-mapk* stable cell line was treated with the indicated dsRNA for 4 d. A rescue effect was observed on both endogenous and exogenous MAPK. (C, F, and G) Densitometry quantifications are provided for MAPK levels (normalized to the AKT loading controls). All experiments were performed in triplicate or more, and *p*-values were calculated (paired two tailed Student’s *t* test) comparing values to the *Rluc* control (*: *p* < 0.05; **: *p* < 0.01). *t* tests performed on other samples are shown in red. “ns” denotes not significant. Numerical data for (A) can be found in S2 Table. Raw data for (C, F, and G) can be found in S1 Data.

We next further narrowed down our list to 13 candidates that displayed a significant rescue effect that was consistent with what we had observed in our primary screen (S4 Table and S1 Text). Of particular interest, *Ubc6*, an E2 ubiquitin-conjugating enzyme, and two E3 ligases, *poe* (*pushover* or *purity of essence*) and *CG5604* (hereafter referred to as *Ufd4* or *Ubiquitin fusion-degradation 4-like*, after the name of its closest yeast counterpart) were identified as the sole E2/E3 enzymes in this set (Fig 5A and 5B). All three factors displayed a capacity to restore normal MAPK levels following *Usp47* RNAi treatment, although *Ufd4* was the weakest (Fig 5A). We were also able to confirm the rescue effects of *poe* and *Ubc6* on endogenous MAPK levels by western blot from S2 cell lysates (Fig 5C) as well as by immunofluorescence in *Drosophila* wing imaginal discs (Fig 5D and S8 Fig). Additionally, *poe* RNAi was able to counteract *Usp47* and restore the ectopic wing vein phenotype induced by *mapk*^*Sem*^ expression in wings (Fig 5E). The ability of POE depletion to restore MAPK levels appeared specific to *Usp47* co-depletion, as demonstrated by *poe* RNAi’s inability to rescue MAPK levels following the depletion of *eIF4AIII*, a regulator of *mapk* splicing (Fig 5F). Moreover, unlike *Uba1*, both *Ubc6* and *poe* did not otherwise seem to have a generalized impact on protein ubiquitination in S2 cells (S9A and S9B Fig), indicating that their activity is restricted to a subset of substrates. Finally, depletion of *poe* and *Ubc6* RNAi also displayed an ability to rescue exogenously expressed HA-tagged MAPK (Fig 5G), which is consistent with a post-translational rescue mechanism.

**Fig 6:**
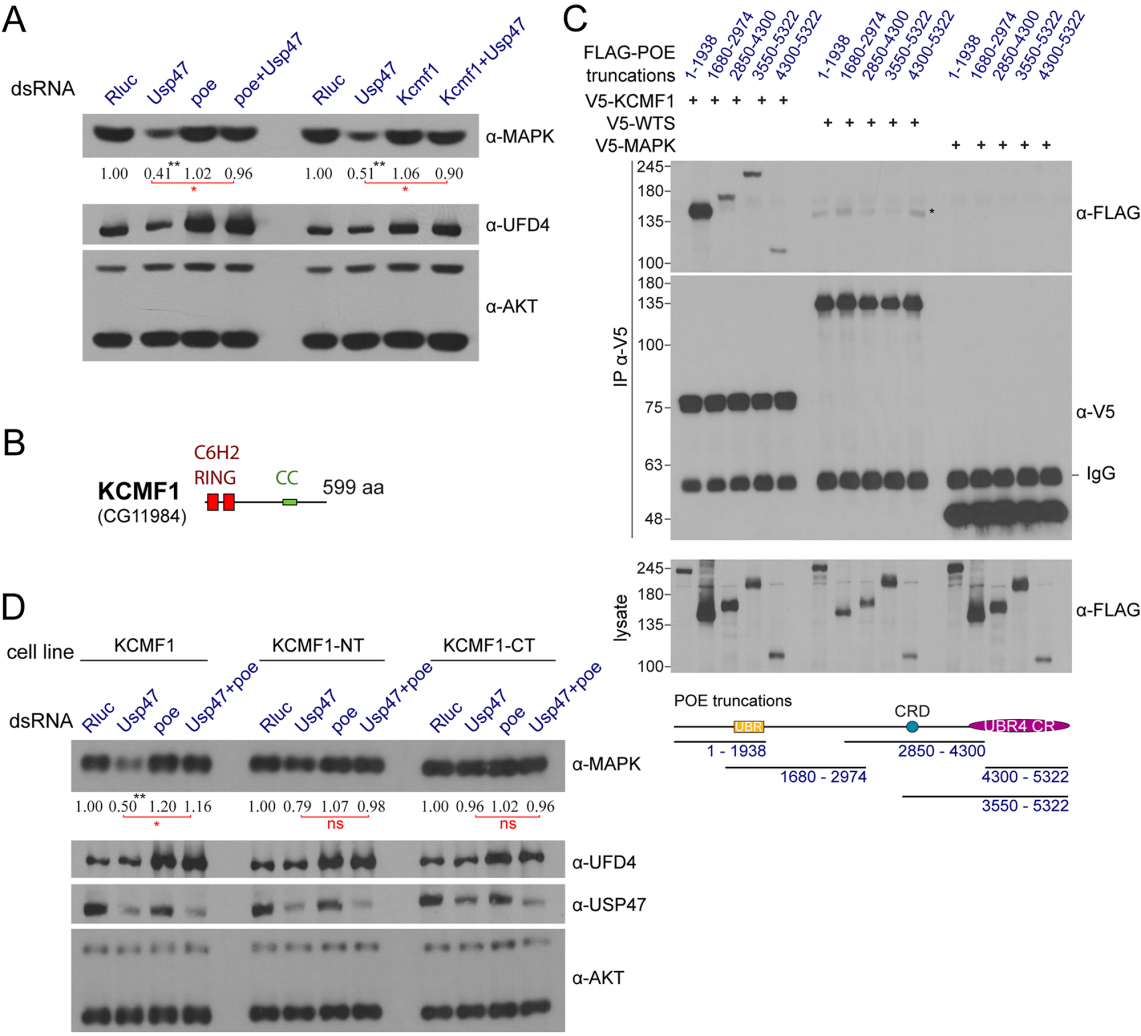
The E3 ligase *Kcmf1* also rescues *Usp47* dsRNA and interacts with *poe*. (A) Western blot displaying that co-depletion of the E3 ligase *Kcmf1* (CG11984) restores MAPK level reduction induced by *Usp47* dsRNA. The impact is similar to that of *poe* co-depletion. Notably, *Ufd4* levels are seen to increase upon *poe* depletion, and this is also observed upon *Kcmf1* depletion (validated by qPCR in S5 Table). (B) Schematic representation of the KCMF1 protein product with the amino acid length and positions of the C6H2-type RING domain (“really interesting new gene”) and predicted coiled-coil region (CC). The C6H2 RING domain is an uncommon zinc-finger subclass that is present in a restricted set of proteins [44] and bears some resemblance to the zinc finger domain of MetAP1 methionine aminopeptidase [45]. (C) Different FLAG-tagged portions of POE (presented in the schematic below the blot with their respective amino acids (a.a.) positions) were expressed in S2 cells and tested for interaction with V5-tagged KCMF1, WTS, and MAPK by immunoprecipitation followed by western blotting. WTS, a member of the HPO (*hippo*) pathway, was used as a negative control. Both truncations containing the UBR4 Conserved Region (UBR4 CR) as well as one truncation comprising the UBR domain and part of POE’s mid region were detected in the KCMF1 IP. By comparison, no interaction was observed between POE and MAPK, while only a faint non-specific band (marked with an asterisk) was observed in the WTS IP. The IgG heavy chain position is also indicated on the α-V5 IP blot. (D) S2 cell lines stably expressing V5-tagged KCMF1 (a.a. 1–599), KCMF1 N-terminal (KCMF1-NT; a.a. position 1 to 239), and C-terminal (KCMF1-CT; a.a. position 241 to 599). KCMF1-NT and CT act as dominant negative proteins and partially rescue MAPK levels following *Usp47* depletion compared to WT KCMF1. KCMF1-NT had a slightly weaker rescue effect than KCMF1-CT. The cell lines were probed by immunoblot following treatment with the indicated dsRNAs. (A and D) Densitometry quantifications are provided for MAPK levels (normalized to the AKT loading controls). All experiments were performed in triplicate or more, and *p*-values were calculated (paired two-tailed Student’s *t* test) comparing values to the *Rluc* control (*: *p* < 0.05; **: *p* < 0.01). *t* tests performed on other samples are shown in red. “ns” denotes not significant. Raw data for (A and D) can be found in S1 Data.

Among the two putative E3 ligases recovered in the screen, *poe* RNAi was the strongest in terms of its capacity to rescue MAPK levels (Fig 5A). In comparison, *Ufd4* depletion produced only a partial rescue effect on MAPK levels (Fig 5A), and this was the case for both dsRNAs tested (S2 Table). However, POE does not contain a readily identifiable E3 ligase domain [21] and is only putatively designated as an E3 because it belongs to the UBR family of E3 ligases, with which it shares a UBR box domain. Because of this, we reasoned that another yet unidentified factor might be acting in conjunction with POE and UFD4. Interestingly, recent work found that UBR4, the human orthologue of POE, physically associates with the zinc-finger protein Potassium Channel Modulatory Factor 1 (KCMF1) [43]. This factor was shown to contain an atypical C6H2-type RING finger domain that may function as a E3 ligase domain [44]. We tested four predicted *Drosophila* orthologues of *Kcmf1*, which were absent from our ubiquitome dsRNA library and thus had not been previously tested for their capacity to rescue the impact of *Usp47* dsRNA on MAPK levels. Strikingly, one of these factors, *CG11984* (hereafter referred to as *Kcmf1* as it is also the closest predicted orthologue to its human counterpart), was found to rescue MAPK levels in a manner that was comparable with *poe* (Fig 6A and 6B). Like *poe* and *Ubc6*, *Kcmf1* knockdown did not have a major impact on global protein ubiquitination in S2 cells (S9A Fig), indicating a similar degree of specificity to MAPK. Moreover, exogenous KCMF1 was found to bind to at least two distinct portions of POE (Fig 6C). Interestingly, knockdown of both *poe* and *Kcmf1* also caused an increase in UFD4 levels (Fig 6A), which provides another indication that these three factors are functionally interrelated. Furthermore, the human orthologues of KCMF1, POE, and UBC6 have recently been shown to co-localize, and the KCMF1 N-terminus was found to bind to UBR4, while the C-terminus associated with human RAD6 (UBC6 counterpart) [43], strengthening the notion that they are functioning as part of an E2-E3 complex. When we stably expressed N- and C-terminal truncations of KCMF1 in S2 cells, we observed a dominant negative suppression of *Usp47* RNAi (Fig 6D), presumably due to the truncated protein segregating its binding partners. In sum, these results show that KCMF1, likely in conjunction with UBC6, POE, and UFD4, plays an important part in destabilizing MAPK and acts in opposition to USP47.

One striking commonality shared by the three factors identified in the *Usp47* genetic interaction screen is that they are all linked to the N-end rule ubiquitin-dependent protein degradation process. The identification of these three factors together thus suggests that MAPK might be the target of N-end rule regulation. This prompted us to test additional N-end rule related factors. Experiments in which we knocked down a series of known and predicted *Drosophila* N-end rule factors by RNAi and assayed their ability to counteract *Usp47* knockdown did not bear fruit, as none of these displayed a capacity to restore MAPK levels (S6 Table). However, upon testing the different *Drosophila* UBR family orthologues by RNAi, we found that the UBR1/2 orthologue also showed a capacity to restore MAPK levels (S10A Fig and S6 Table). Unlike POE/UBR4, the UBR1/2 orthologue caused a significant reduction in cell count (S6 Table), explaining why it was not retained in our *Usp47* genetic interaction screen. Thus, this additional result lends further support to the notion that MAPK might be the target of an N-end rule mechanism.

Since a majority of proteins undergo co-translational cleavage of their initiator methionine by methionine aminopeptidases [46], it is possible that the glutamate following the methionine of MAPK might be exposed, which could constitute an N-degron. Most of our experiments in which MAPK is expressed exogenously make use of an N-terminally tagged HA-MAPK. As HA-MAPK still responds to *Usp47* RNAi, this suggests that the glutamate residue that follows the N-terminal methionine on WT MAPK is not involved in regulation by USP47. To further investigate this possibility, we employed the ubiquitin-fusion technique, which allows for selective exposure of a residue at the N-terminus of a peptide [47], and used this method to expose the penultimate glutamate residue on MAPK (S10B Fig). However, both the N-terminally exposed glutamate (WT) and glycine (stabilizing mutant) forms were sensitive to *Usp47* depletion, indicating that *Usp47* does not intervene on the N-terminal penultimate residue of MAPK (S10C and S10D Fig). This also precludes the possibility that the initiator methionine itself might constitute an N-degron (recent work has shown that when the initiator methionine is not cleaved, N-acetylation of the methionine can also constitute an N-degron [19]) as MAPK, without its N-terminal methionine, still responded to *Usp47* RNAi (S10D Fig).

Thus, our results suggest that MAPK turnover is regulated by an N-end rule-mediated process and that USP47 counteracts the destabilizing impact of these factors on MAPK. However, it remains unclear whether or not MAPK is a direct target of this process, and, in the event that it is a direct target, it appears that neither the N-terminal methionine nor the glutamate that follows it is involved in recognition by the degradation machinery.

## Discussion

**Fig 7:**
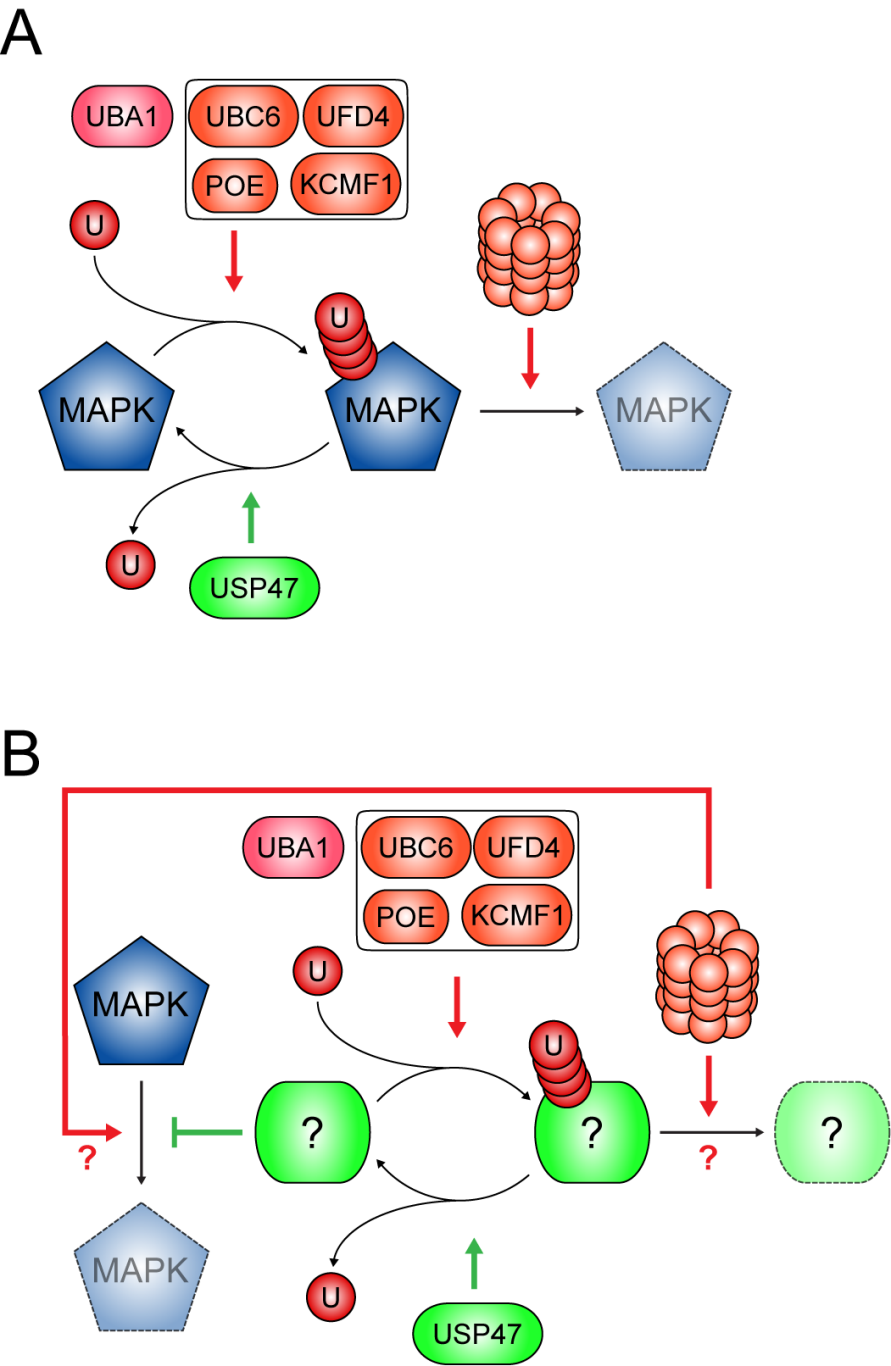
Models for regulation of MAPK stability by USP47. (A) Direct ubiquitination of MAPK. In this model, UBC6, POE, UFD4, and KCMF1 would participate in the direct ubiquitination of MAPK. Ubiquitinated MAPK could then be targeted to the proteasome for degradation. In the presence of USP47, MAPK would be stabilized as USP47 acts to deubiquitinate MAPK, thus counteracting the activity of the ubiquitin ligases. (B) Indirect regulation of MAPK stability. In this alternative model, UBC6, the E3s, and USP47 act on a yet unidentified factor whose proposed role is to stabilize MAPK. In this scenario, the unidentified factor is the direct target of (de)ubiquitination, whereas MAPK is destabilized through ubiquitin-independent means. The unidentified factor may be degraded by the proteasome following its ubiquitination. MAPK might be degraded through a process that does not implicate the proteasome. Alternatively, even though it is not directly ubiquitinated, MAPK might be degraded by the proteasome through ubiquitin-independent degradation. One possibility in this latter case would be that the unidentified factor acts as a chaperone, bringing MAPK to the proteasome. (A,B) Arrow colors are used to indicate positive (green) or negative (red) regulatory impact on MAPK.

In this study, we show that USP47, which was previously identified in a genome-wide screen for factors acting downstream of RAS^V12^ [8], acts to stabilize MAPK post-translationally through a mechanism involving the UPS. We also present results from a new candidate-based RNAi screen for genetic interactors of *Usp47* that led us to identify three new factors that act to destabilize MAPK and thus counteract USP47 activity. Moreover, we present evidence for in vivo regulation of MAPK levels and signaling by USP47, POE, and UBC6 that corroborates our cell culture data. Lastly, we identify a fourth destabilizing factor, the putative E3 ligase KCMF1, and demonstrate that it acts in a similar manner to POE. Together, these results present strong evidence for a new means of post-translational control of MAPK levels (Fig 7), which unravel a completely novel aspect of MAPK regulation.

The screening approach we employed in this study differs from classical techniques in that it was based on a genetic interaction approach by RNAi co-depletion. This approach is reminiscent of synthetic genetic interaction screens commonly conducted in yeast, but has only recently been introduced to the RNAi screening field [39,40]. Our results further support this approach as a viable technique in identifying functionally related factors, i.e. factors counteracting *Usp47*, in this case. The advantage of such co-depletion-based approaches is highlighted by the fact that the ubiquitin-proteasome components and ligases identified here were not identified in the initial RAS-MAPK pathway screen that uncovered *Usp47*. This can be readily explained by our observation that these factors only have a sizeable impact on MAPK levels in a context in which *Usp47* is depleted. Thus, while the regulatory landscape of the RAS-MAPK pathway has now been extensively explored through single-depletion RNAi screening [8,48], it is likely that other regulatory components would be uncovered by further co-depletion/genetic interaction RNAi screens.

The most striking result from our *Usp47* co-depletion RNAi screen was the identification of the three N-end rule related factors, *poe*, *Ubc6*, and *Ufd4*. In contrast to the canonical N-recognins UBR1 and UBR2, non-canonical N-recognins such as UBR4 remain relatively poorly studied. UBR4 in particular is characterized by the absence of a readily identifiable ubiquitin ligase domain, which may indicate that they function by associating with catalytically factors such as UBR1/2, UFD4 or possibly KCMF1. *CG5604* is referenced as *Ufd4* in this study because it is the fly gene with highest sequence homology to *Saccharomyces cerevisiae* UFD4, a HECT domain ubiquitin ligase that is a member of the ubiquitin fusion degradation (UFD) family (Fig 5B). The UFD pathway is specifically linked to the recognition and ubiquitination of fusion proteins that incorporate a N-terminal ubiquitin moiety [49]. UFD4 in particular has been shown to recognize the N-terminal ubiquitin moiety of UFD substrates via its armadillo repeats [50]. Interestingly, UFD4 (and other UFD pathway factors) has also been shown to work synergistically with the N-end rule pathway [23] and to interact physically and functionally with UBR1 and contribute to the ubiquitination and degradation of N-end rule substrates [51]. Intriguingly, UBC4 (the main E2 associated to UFD4) was not found to act on MAPK levels, indicating that if there is such a UBR4-UFD4 complex, it most likely would operate differently from the UBR1/RAD6-UFD4/UBC4 complex described in yeast.

One alternative to the N-end rule pathway hypothesis is that these factors instead control lysosomal proteolysis of MAPK. Indeed, recent studies have linked RAD6, UBR4, and KCMF1 to lysosomal degradation and autophagy. UBR4 has both been shown to be degraded through autophagy and to have an impact on autophagic flux in mice [52]. RAD6 has been shown to promote mitochondrial turnover (mitophagy) in both flies and mammals [53] and also to promote the autophagic degradation of HP1, a heterochromatin factor involved in DNA repair [54]. More recently, RAD6, UBR4, and KCMF1 have been found to interact physically and to bind lysosomal/mitophagy factors as well [43]. However, based on our data, a hypothetical autophagy-based mechanism for MAPK degradation would also have to incorporate proteasomal regulation, invoking a model involving both systems. To test this possibility, we assayed some of the principal autophagy-related factors by RNAi, but observed no impact of MAPK levels (S6 Table), and this was also the case for predicted RAD6-associated factors from Hong et al. (S6 Table) [43]. Thus, despite previously published links, our data does not support an autophagy/lysosome-based degradation mechanism for MAPK.

USP47 does not uniquely work on MAPK, as it has been found to stabilize other proteins. For instance, both the human and *Drosophila* forms have recently been found to stabilize β-catenin [17]. Human Usp47 has also been shown to be transported to adherens junctions by the KIFC3 kinesin, where it then acts to stabilize E-cadherin [16]. In flies, *Usp47* has also been previously described as a negative regulator acting downstream of MAPK in the context of eye development, in which it stabilizes the transcriptional repressor TTK [10]; our description of *Usp47* as a positive regulator seemingly opposes this previously described role and implies that *Usp47* may play a dual role in the RTK/MAPK pathway with respect to eye development. Importantly, another E3 ligase, SINA, was implicated in the context of TTK, indicating a difference in the way these two components are regulated. This would explain the different outcomes we obtained in our genetic interaction experiments (Fig 1D and S1 Fig and [10]); specific genetic lesions in the MAPK pathway may be more sensitive to the positive or negative impact of regulation by USP47. One plausible model is that USP47 inactivation leads to a rapid degradation of TTK followed by a slower degradation of MAPK. Thus, inactivating USP47 could initially provide a positive input into the pathway as TTK levels drop rapidly, which would then transition into a negative input as MAPK levels gradually decrease.

Our findings indicate that USP47, together with UBC6, POE, UFD4, KCMF1, and the UPS, is part of a regulatory process that acts to control MAPK levels post-translationally. This discovery was unexpected given that MAPK was not generally known to be regulated by the UPS. Two notable exceptions to this deserve mention. Firstly, human ERK1/2 has been observed to undergo proteasomal degradation in a hyperosmotic condition induced by a prolonged treatment with high levels of sorbitol [55]. Secondly, ERK1c, an alternatively spliced isoform of ERK1, has been shown to accumulate in the Golgi apparatus and undergo monoubiquitination in conditions of elevated cell density [56]. However, to our knowledge, the UPS was not previously known to regulate the stability of the main isoforms of MAPK (ERK1/2) in physiological conditions. The impact of the UPS on MAPK may have eluded previous investigation attempts due to the presence of USP47, which stabilizes MAPK in basal conditions. Indeed, it is only in USP47-depleted conditions that we were able to observe the effect of ubiquitin/proteasome components on MAPK. Furthermore, our inability to observe direct ubiquitination of MAPK may constitute another reason why this phenomenon had eluded prior investigation. It is possible that MAPK is subjected to a non-canonical form of ubiquitination [57] that is more difficult to detect or that it is altogether not the direct target for ubiquitination and is instead degraded through a mechanism that relies on unstructured regions or a ubiquitinated chaperoning protein for targeting to the proteasome (Fig 7B) [57,58].

Future work examining the regulation of USP47 and the associated ubiquitin ligases will be needed to determine in what physiological context this system is used to control MAPK levels. In terms of our understanding of pathway function, these findings add another layer of regulation to the set of transcriptional and splicing regulators we have described previously [7,8], thereby positioning MAPK at the center of an intricate web of regulators acting on its expression. Further exploration of the environmental or developmental cues that control these factors will no doubt have important implications for our understanding of RAS-MAPK signaling dynamics.

## Materials and Methods

### Nomenclature

Following the recommended *Drosophila* nomenclature, gene names are italicized. Protein product names are not italicized and, with the exception of p38, are capitalized to distinguish from gene names. *CG5604* is referred to here as *Ufd4* based on the name of its predicted yeast orthologue and the naming scheme used for other UFD family members in *Drosophila* such as *Ufd1-like* (FBgn0036136). *CG11984* (FBgn0037655) is referred to as *Kcmf1* based on the name of its human counterpart. Upon first mention of a specific *Drosophila* gene or protein, the FlyBase gene ID number is provided. The FlyBase gene symbol is also listed when it differs from the symbol used in this text.

### RNAi

All dsRNAs were generated by in vitro transcription using T7 RNA polymerase. Following NaOAc ethanol precipitation, dsRNA concentration was assessed by gel dosage. Individual dsRNA probes were added to cells to a final concentration of 10 μg/ml. RNAi in S2 cells was conducted using dsRNA following previously described procedures [8]. For the *Usp47* co-depletion screen and other quantitative microscopy-based experiments, cells were pre-incubated with *Usp47* or GFP control dsRNA for 3 d and then distributed in clear-bottom 384 well plates (Greiner) containing the dsRNA sets for a second 3 d depletion. This was done as *Usp47*’s impact on MAPK levels was strongest at incubation times greater than 3 d, whereas other dsRNAs can cause lethality at longer depletion times. The full list of dsRNA reagents and primer sequences used in this study is presented in S7 Table. Subsequent to the *Usp47* candidate screen, we selected one of the two dsRNAs from the validation step to use for follow-up experimentation. See S1 Text for further information on the targeted USP RNAi library.

### RT-qPCR and RT-PCR

RT-qPCR was conducted using TaqMan Gene-specific assays (primer sets and TaqMan hydrolysis probes), which were designed using the Universal Probe Library assay design center (Roche Applied Science). Primer sequences and the Universal Probe Library probe number are listed in S7 Table. Assays were designed such that the amplified regions did not overlap with sequences targeted by dsRNA. *Usp47*, *Uba1*, *Kcmf1*, and hit validation qPCR experiments were performed on S2 cell lysates obtained using the Cells-to-cDNA (Ambion) lysis buffer following treatment with the indicated dsRNAs. Samples were biological triplicates (validation and *Usp47*), quadruplicates (*Uba1* and *Kcmf1*), or six biological replicates (polysome RNA extraction). The in vivo experiments were biological triplicate samples prepared from the brain and eye/antennal disc tissue of four L3 larvae per sample. Samples from the polysome fractionation experiment were prepared as previously described [7], after which RNA extraction was performed using TRIzol reagent (Invitrogen).

With the exception of the polysome fractionation experiment in which Ct values are presented, all other qPCR values were log normalized to negative control samples (*GFP* dsRNA for the cell culture dsRNA experiments, or *mCherry* RNAi controls in the case of the in vivo experiments). *Act5C* and *RpL32* (FBgn0002626) were used as reference genes for standardization. Student’s *t* tests (homoscedastic, two-tailed) were performed by comparing the experimental values to those of the negative control samples.

For RT-PCR, 5 μg of total RNA extracted from S2 cells (RNeasy, Qiagen) was primed with random primers followed by reverse transcription (RT) with SuperScript II Reverse Transcriptase (Invitrogen). Primers are also listed in S7 Table.

### High-Content Microscopy

RNAi-treated S2 cells in 384 well plates were fixed in 4% paraformaldehyde and stained overnight with an α-MAPK antibody (1/1000, Cell Signaling #4695), phalloïdin, and DAPI. Plates were imaged on an Operetta (PerkinElmer) imaging platform. Acquired images were segmented and analyzed using Harmony (PerkinElmer) to derive the average MAPK and actin levels per cell as well as the average cell count per well.

### Cell Culture, Western Blots, Immunoprecipitation, and Metabolic Labeling

S2 cell culture, transfection, lysates, and western blotting were performed as previously described [59]. Pulse-chase radioactive labeling was performed by incubating S2 cells in ESF medium containing [35S]-methionine for 6 h. Cells were then harvested at different time points (0–36 h) following this incubation period, after which an α-HA immunoprecipitation was performed to enrich for HA-MAPK. Fluorographic reagent (NAMP100, GE Healthcare) was used to amplify [35S] signal. To visualize newly translated MAPK, we used a shorter labelling period of 4 h, after which cells were immediately lysed and submitted to immunoprecipitation. In both metabolic labeling experiments, RNAi treatment was performed for 5 d at a concentration of 15 μg/ml. Epoxomicin treatment was performed at a concentration of 1 μM (DMSO used for controls) 18 h prior to lysis of S2 cells. Whole fly lysates were prepared from 20 adults homogenized in 500 μl of RIPA buffer.

### Fly Genetics and Microscopy

Fly husbandry was conducted according to standard procedures. All crosses were performed at 25°C. The *Usp47*^Δ*1*^, *Usp47*^Δ*2*^, and *Usp47*^*rev*^ lines were initially described in [10]. The *rl*^*1*^ (FBst0000386) and *UAS-rl*^*Sem*^ (FBst0059006) lines were obtained from Bloomington. The *sal*^*EPv*^*-Gal4* line was a kind gift from J.F. de Celis, and the *csw*^*lf*^ line was originally obtained from L. Perkins. RNAi clones were generated using a line carrying a heat shock inducible flip-out actin promoter driving the expression of GAL4 and GFP in clonal tissues (*hs-flp;; Act5C*.*CD2*.*Gal4*, *UAS-GFP*). L1 larvae were heat shocked for 15 min at 37°C and later collected for dissection upon reaching late L3 (wandering) stage. RNAi expression in wing discs was carried out using an *engrailed-Gal4* driver line also carrying *UAS-dcr2* to enhance RNAi depletion effectiveness. Staining was performed as previously described [8], and immunofluorescence confocal microscopy was conducted using a Zeiss LSM 700. For the in vivo qPCR experiments, RNAi expression was induced by a prolonged 30' heat shock using *hs-flp;; Act5C*.*CD2*.*Gal4*, *UAS-GFP* flies to express the indicated RNAi constructs in large clones that encompassed most of the larval tissue. *Ubc6* RNAi expression was more problematic as the RNAi caused larval lethality, and clonal expression was not as prominent in tissue recovered from escapers. Accordingly, qPCR results for *Ubc6* showed a weaker and more variable depletion.

## Supporting Information

S1 Data Underlying data and quantitative observations presented in figures. (XLSX)

S1 Fig CKDN and EGFR genetic interaction experiments. Two hypomorphic alleles of *Usp47* (*Usp47*^*Δ1*^ and *Usp47*^*Δ2*^) were tested for genetic interaction with *bona fide* RAS/MAPK pathway mutants. Both the *Usp47*^*Δ1*^ and *Usp47*^*Δ2*^ alleles are caused by imprecise excisions of the *P*(*EP*)*GE28938* P element and the *Usp47*^*rev*^ (revertant) control was generated by precise excision of the same P element [10]. (A) *Usp47*^*Δ1*^ and *Usp47*^*Δ2*^ were found to enhance the severity of the rough eye phenotype in flies homozygous for *rl*^*1*^, a hypomorphic allele of *mapk*. MAPK signaling is impaired in *rl*^*1*^ homozygotes, leading to a mild rough eye phenotype that is due to a lack of photoreceptor cells. Flies homozygous for both *Usp47* alleles (only *Usp47*^*Δ2*^ is shown here) also present a slight rough eye phenotype as has been previously reported [10]. Flies homozygous for both the *mapk* and the *Usp47* mutations display an increase in the severity of the rough eye phenotype, which is consistent with a positive role of USP47 in MAPK signaling. (B) The corkscrew (*csw*) phosphatase is a positive regulator of RAS/MAPK signaling acting upstream of RAS. Like *mapk*^*1*^ flies, hemizygotes for a *csw* loss of function (*csw*^*lf*^) mutation also have an impaired RAS/MAPK pathway, which translates itself in a visible rough eye phenotype. In flies bearing both *csw*^*lf*^ and homozygous for either *Usp47*^*Δ1*^ and *Usp47*^*Δ2*^, the severity of the rough eye is also increased, while this collaboration was not observed for the *Usp47*^*rev*^ control.
(TIF)

S2 Fig Structural and functional determinants of MAPK regulation by USP47. (A) S2 cell lines stably expressing HA-MAPK, HA-JNK, or HA-p38B were treated with *Usp47* dsRNA. Unlike HA-MAPK, HA-JNK and HA-p38B levels do not appreciably respond to *Usp47* dsRNA. (B) pMAPK levels were probed following transfection of constitutively active MEK (MEK^EE^) in S2 cells stably expressing wild-type exogenous HA-tagged MAPK or the mutants described in Fig 3C, in which either external lysines (MAPK^KextR^) or all lysines (MAPK^noK^) were switched to arginines. MAPK^noK^ does not appear to be phosphorylatable by MEK^EE^. (C) Stably expressed MAPK mutants altering the proper folding of MAPK partially negate the impact of USP47 depletion. The L41E mutation is located in the kinase’s N-lobe, in the β1 strand preceding the glycine-rich ATP-phosphate-binding loop. The W225E is located in the αF helix that follows the activation segment and which forms the core of the C-lobe. Introduction of a charge residue (Glu) within these two key structural elements disrupts hydrophobic interactions and, hence, alters the structural integrity of the kinase domain. (D) Truncation of MAPK C-terminal tail also partially abrogates its sensitivity to USP47 depletion. Truncated MAPK was stably expressed in S2 cells and treated with the indicated dsRNAs. The Y329* truncation removes the C-terminal extension of MAPK, which includes helix α_L16_ that associates with the N-lobe. (E) The T and Y residues of MAPK’s activation segment are not required for regulation by *Usp47*. The TEY residues of MAPK’s activation segment at position 198–200 were replaced with AEF and stably expressed in S2 cells. Stably expressed HA-tagged wild-type MAPK is used as a control. (F) The ERK1/2 family D-site recruitment site that interacts with the docking motif of substrates and other ERK/MAPK interactors is not required for regulation by *Usp47*. The D-site recruitment site of *Drosophila* MAPK was inactivated by changing the D331 and D334 residues to asparagines. Exogenous MAPK modified in this manner was stably expressed and submitted to treatment with *Usp47* dsRNA. (G) Samples prepared from stable *Drosophila* S2 cell lines expressing HA-tagged mouse ERK1 and human ERK2 or *Drosophila* HA-MAPK were treated with the indicated dsRNA. Western blotting shows that, like *Drosophila* MAPK, ERK1/2 also decrease following *Usp47* knockdown. AKT is used as a loading control. (A, C–E, and G) Densitometry quantifications are provided for MAPK levels (normalized to the AKT loading controls). All experiments were performed in four replicates or more, and *p*-values were calculated (paired two-tailed Student’s *t* test) comparing values to the *Rluc* control (*: *p* < 0.05; **: *p* < 0.01). *t* tests performed on other samples are shown in red. “ns” denotes not significant. Raw data for (A, C–E, and G) can be found in S1 Data.
(TIF)

S3 Fig Ubiquitination of MAPK is not detectable by immunoprecipitation of HA-tagged ubiquitin. V5-tagged MAPK, KSR, and RAF were expressed alone or co-transfected with HA-tagged ubiquitin. Following α-V5 immunoprecipitation, samples were probed with α-HA to reveal polyubiquitinated forms (right panel). The α-V5 IP levels are shown in the middle panel, and lysates probed with α-V5 are shown in the left panel.
(TIF)

S4 Fig Newly synthesized MAPK is not sensitive to *Usp47* depletion. (A) S2 cells were treated with *Rluc*, *mapk*, or *Usp47* dsRNA for 5 d, then were subjected to metabolic labeling with [35S]-methionine for 4 h. Radio-labeled (newly synthesized) MAPK was immunoprecipitated and detected by fluorography following western blotting. (B) Steady-state levels of MAPK from the samples shown in (A) were determined by western blotting to confirm their reduction upon USP47 depletion. Endogenous AKT is used as a loading control.
(TIF)

S5 Fig Evaluating genetic interactions between two dsRNAs using a quantitative phenotype. (A) Principle for defining a genetic interaction based on quantitative trait values obtained from single depletion of gene *a* and *b* as well as combined depletion. If the combined result is purely additive, there is no genetic interaction (Δ*m* = 0). An aggravating genetic interaction takes place when the combined depletion value increases the severity of the phenotype beyond the expected additive value (this is also called positive epistasis; Δ*m* < 0). Conversely, an alleviating effect takes place when the combined depletion is less severe than the expected additive value (negative epistasis; Δ*m >* 0). The Δ*m* is further explained in S1 Text. (B and C) Hypothetical genetic interaction examples in which *a* is replaced by *Usp47* RNAi. Since *Usp47* KD has a negative impact on MAPK levels, the Δ*m* for alleviating and aggravating interactions will be of the opposite sign to those associated with gene *a* KD in (A). (B) In this example, an RNAi targeting gene *x* reduces MAPK levels. A purely additive result for *Usp47* + *x* indicates that no genetic interaction is taking place and that gene *x* will slightly reduce MAPK levels irrespective of whether *Usp47* is depleted or not. This might occur if a transcription or splicing factor that acts on *mapk* were to be co-depleted with *Usp47*; like *Usp47*, the factor also reduces MAPK levels, but functions in parallel, acting at a completely different regulatory step. However, if *x* aggravates the impact of *Usp47* RNAi by decreasing MAPK levels beyond the expected additive value, then *x* interacts genetically with *Usp47*. An example of this would be a putative deubiquitinase acting redundantly with *Usp47*. On the contrary, an alleviating genetic interaction takes place if *Usp47*+*x* is lesser than that expected additive value. This might occur in the case of a factor acting in the same “pathway” as *Usp47*, for instance, a transcription factor promoting *Usp47* expression or a factor stabilizing USP47 protein levels. (C) An RNAi targeting gene *y* slightly increases MAPK levels. The additive value of *Usp47 + y* (marked with an arrowhead) is represented here by subtracting the empty *y* bar (dashed line contour) from the full black *Usp47* bar. If *y* alleviates the impact of *Usp47* RNAi by restoring MAPK levels beyond this expected additive value, then *y* interacts genetically with *Usp47*. An example of this would be a putative ubiquitin ligase or UPS component. This category of hit was most frequently observed in our screen. An aggravating effect in this example is more counterintuitive, invoking a mechanism whereby RNAi of *y* has a positive impact on MAPK when depleted alone, but has an opposite effect—causing an enhanced reduction of MAPK levels—when combined with *Usp47* dsRNA. A factor that targets MAPK to a cellular compartment where it is protected from both USP47 and any potential DUBs might be a good example of an aggravating genetic interactor in this case.
(TIF)

S6 Fig Additional data and hit selection strategy for the *Usp47* genetic interaction RNAi screen. (A) MAPK levels from the *Usp47* RNAi screen are plotted for *GFP* co-depletion samples against the corresponding *Usp47* co-depletion samples. *X*-axis values are normalized to *GFP* dsRNA controls, while *y*-axis values are normalized to *Usp47* dsRNA controls. In general, MAPK levels from *Usp47* co-depletion samples generally mirror those of the *GFP* co-depletion controls (following the dashed diagonal). This suggests that most candidates do not differ from the expected neutral phenotype of a merely additive co-depletion effect. Genetic interactors of *Usp47* are situated either above the diagonal (alleviating: green shaded area) or below the diagonal (aggravating: red shaded area). Ubiquitin genes, *poe*, *Ubc6*, and *Ufd4*, are highlighted as examples of factors that alleviate *Usp47* RNAi. (B) Volcano plot of Δ*m* scores plotted against Δ*m p*-values. The two values are generally well correlated, prompting us to use the *p*-value to guide candidate selection. (C) Average cells per well imaged for both *GFP* and *Usp47* co-depletion samples throughout the screen. The absence of a significant difference between the two suggests that there was not a widespread synthetic lethality effect caused by co-depletion of *Usp47* and other ubiquitin-proteasome factors. (D) A reduction in cell count is correlated with a non-neutral Δ*m* (***: *p* < 0.001). We therefore used cell count as a secondary criterion to increase the stringency of hit selection. (E) Graphical representation of the hit selection criteria. The false discovery rate (FDR) of Δ*m* is plotted against the FDR of the cell count values. A global Δ*m* cutoff (blue line) is used to select hits irrespective of their impact on viability (blue shaded area). A second less stringent Δ*m* cutoff (red line) is used to select candidates that had little or no impact on cell count (red shaded area). Raw data for (B-D) can be found in S1 Data. Data related to (A and E) is contained in S1 Table.
(TIF)

S7 Fig DUB redundancy screen. Results from the DUB co-depletion screen. *Usp47* RNAi depletion has a partial impact on MAPK levels, even at prolonged depletion times. An independent set of dsRNAs targeting the predicted DUBs in *Drosophila* was designed to specifically re-screen these genes to look for any potential redundant factors that might act synergistically with *Usp47*. However, as was the case in the primary screen, no other DUB was found to cause an appreciable MAPK reduction, either on its own or in conjunction with *Usp47* depletion. *Rpn11* does, however, rescue MAPK levels in co-depletion with *Usp47*. This is most likely attributable to its function as a proteasome component. The data used to prepare this figure is contained in S3 Table.
(TIF)

S8 Fig In vivo depletion of *poe* and *Ubc6* alone has no impact on MAPK levels. As a control for the dual RNAi depletions presented in Fig 5D, we depleted *poe* and *Ubc6* alone, using the same RNAi lines. The *engrailed-gal4* driver was used to drive expression of RNAi in the posterior segment (GFP-positive) of the disc. *Ubc6* RNAi expression caused extensive larval lethality. Those wing discs that could be recovered were of reduced size. The RNAi lines used in this experiment correspond to the following VDRC lines: *mapk*, KK109108; *poe* (1), KK108296; *poe* (2), GD17648; *Ubc6*, GD23229. Canton-S flies were crossed to *engrailed-gal4* flies as a negative control.
(TIF)

S9 Fig *Ubc6*, *Ufd4*, *poe*, and *CG11984* knockdown do not have a major impact on global protein ubiquitination in S2 cells. (A) S2 cells were treated with the indicated dsRNA and ubiquitin was subsequently examined by western blot (duplicate samples). USP47 and the E2/E3 ligases acting on MAPK did not visibly alter global protein ubiquitination. (B) *Uba1* and *pros α5* dsRNAs were used as positive controls for factors causing ubiquitin reduction and accumulation, respectively.
(TIF)

S10 Fig Additional data on N-end rule and MAPK. (A) We retested the *Drosophila* UBR family factors (N-recognins) to verify whether members of this family other than UBR4/POE also suppress the impact of *Usp47* dsRNA on MAPK levels. Independent dsRNAs were designed for *UBR1/2* (*CG9086*), *UBR3* (*CG42593*), *UBR4* (*poe*), *UBR5* (*hyd*), *UBR6* (*FBX011*), and *UBR7* (*CG15141*). Besides *UBR4/poe*, only *UBR1/2* dsRNA had a robust alleviating impact on MAPK levels when co-depleted with *Usp47* (***: *p* < 0.001; ****: *p* < 0.0001). However, unlike *UBR4*, *UBR1/2* RNAi caused a sharp reduction in cell count (see experimental data in S6 Table). (B) Diagram of the ubiquitin-fusion construct used to expose the glutamate residue following the initiator methionine (penultimate residue). Ubiquitin is cleaved by DUBs (following the G76 residue), exposing the penultimate residue. (C) The Ub-fusion MAPK protein is cleaved to expose the penultimate residue (the glutamate residue following the initiator methionine on WT MAPK). An uncleavable G76V Ub mutant is used as a control for co-translational Ub cleavage. The E2G MAPK mutant has the penultimate glutamate replaced with a glycine (a stabilizing residue). For this experiment, S2 cells were transiently transfected with the Ub-fusion constructs prior to treatment with epoxomicin or DMSO (controls). (D) Following cleavage of the N-terminal ubiquitin, both the WT (glutamate) and E2G mutant forms of MAPK are sensitive to *Usp47* depletion. S2 cells were stably transfected with a Ub-fusion construct containing either the WT MAPK sequence (WT) or the E2G mutant form. These cell lines were then treated with *Usp47* dsRNA for 4 d. Two exposures of the MAPK blot are presented as differences in expression levels of the Ub-MAPK fusions between both cell lines make it difficult to observe the *Usp47* depletion effect on a single exposure.
(TIF)

S1 Table Primary screen results. (XLSX)

S2 Table RNAi validation screen results. (XLSX)

S3 Table DUB screen results. (XLSX)

S4 Table Validated hits list. (PDF)

S5 Table qPCR validation of selected candidate dsRNAs. (XLSX)

S6 Table Additional RNAi experiment data. (XLSX)

S7 Table Primer information for dsRNAs, RT-PCR, and RT-qPCR assays. (XLSX)

S8 Table Fly genotypes. (XLSX)

S1 Text Additional materials and methods. (DOCX)

## Abbreviations

BER: base-excision repair
DRS: D-site recruitment site
DUB: deubiquitinase
ERAD: endoplasmic-reticulum-associated protein degradation
FRS: F-site recruitment site
mRNP: messenger ribonucloeprotein
RTK: receptor tyrosine kinase
UBR: ubiquitin protein ligase E3 component n-recognin
UFD: ubiquitin fusion degradation
UPS: ubiquitin proteasome system
USP: ubiquitin specific protease
