# Supplementary figures and images for: The Deubiquitinase USP47 Stabilizes MAPK by Counteracting the Function of the N-end Rule ligase POE/UBR4 in Drosophila
Source: PLoS Biol. 2016 Aug 23;14(8):e1002539. doi: 10.1371/journal.pbio.1002539 (PMC4994957; doi:10.1371/journal.pbio.1002539)

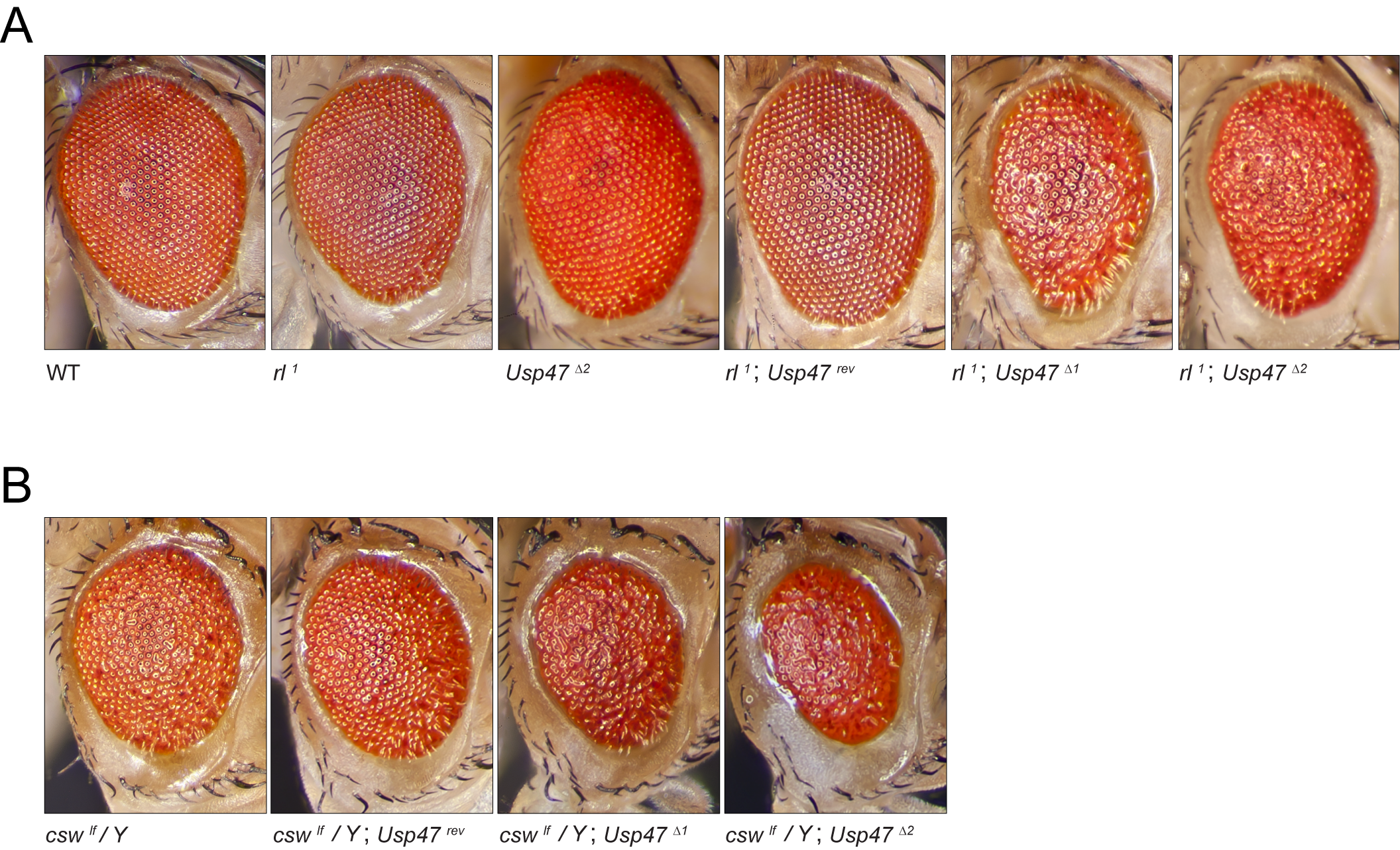

Supplement: S1 Fig — Two hypomorphic alleles of Usp47 (Usp47Δ1 and Usp47Δ2) were tested for genetic interaction with bona fide RAS/MAPK pathway mutants. Both the Usp47Δ1 and Usp47Δ2 alleles are caused by imprecise excisions of the P(EP)GE28938 P element and the Usp47rev (revertant) control was generated by precise excision of the same P element [10]. (A) Usp47Δ1 and Usp47Δ2 were found to enhance the severity of the rough eye phenotype in flies homozygous for rl1, a hypomorphic allele of mapk. MAPK signaling is impaired in rl1 homozygotes, leading to a mild rough eye phenotype that is due to a lack of photoreceptor cells. Flies homozygous for both Usp47 alleles (only Usp47Δ2 is shown here) also present a slight rough eye phenotype as has been previously reported [10]. Flies homozygous for both the mapk and the Usp47 mutations display an increase in the severity of the rough eye phenotype, which is consistent with a positive role of USP47 in MAPK signaling. (B) The corkscrew (csw) phosphatase is a positive regulator of RAS/MAPK signaling acting upstream of RAS. Like mapk1 flies, hemizygotes for a csw loss of function (cswlf) mutation also have an impaired RAS/MAPK pathway, which translates itself in a visible rough eye phenotype. In flies bearing both cswlf and homozygous for either Usp47Δ1 and Usp47Δ2, the severity of the rough eye is also increased, while this collaboration was not observed for the Usp47rev control. (TIF) [file pbio.1002539.s002.tif]

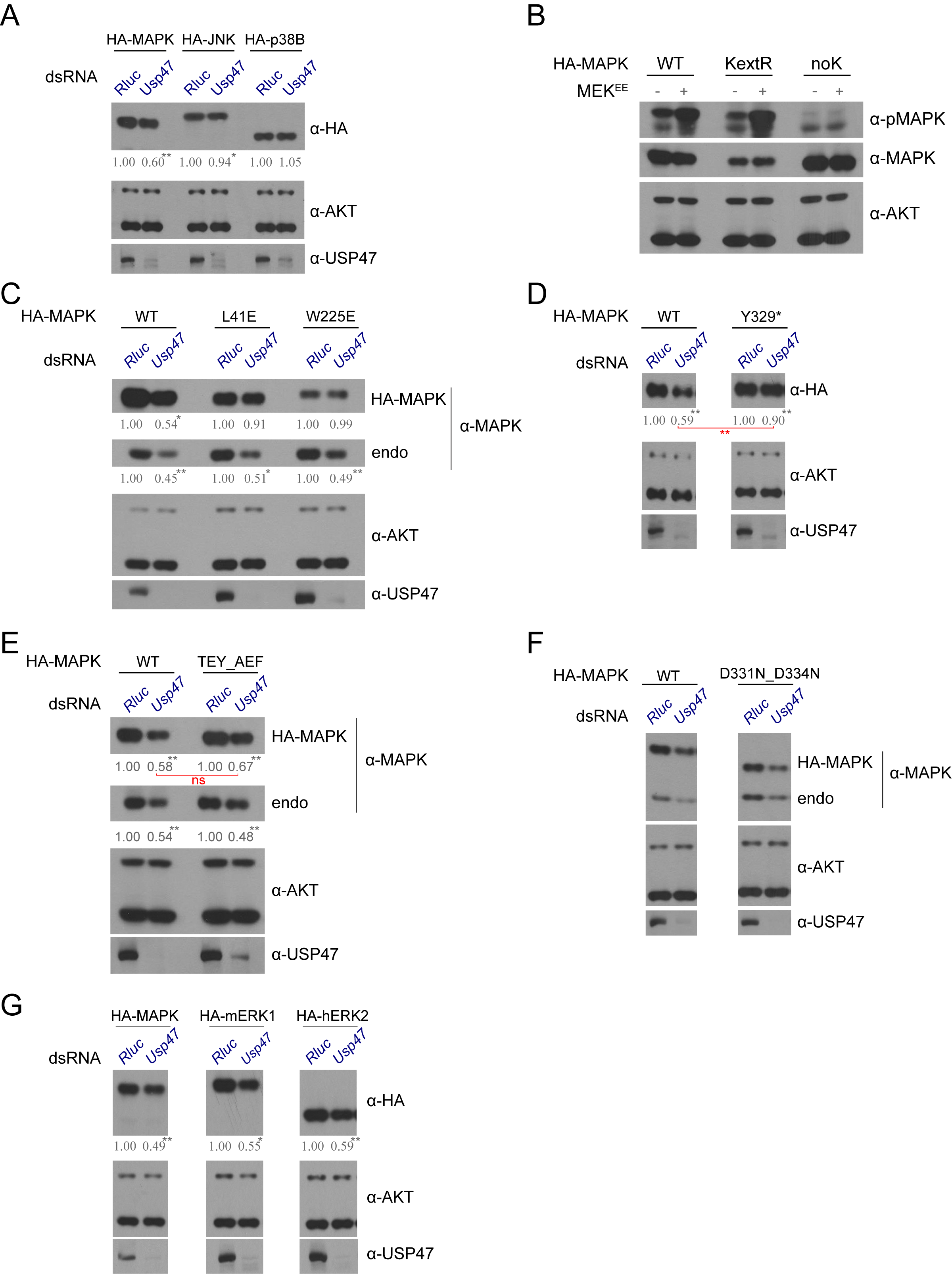

Supplement: S2 Fig — (A) S2 cell lines stably expressing HA-MAPK, HA-JNK, or HA-p38B were treated with Usp47 dsRNA. Unlike HA-MAPK, HA-JNK and HA-p38B levels do not appreciably respond to Usp47 dsRNA. (B) pMAPK levels were probed following transfection of constitutively active MEK (MEKEE) in S2 cells stably expressing wild-type exogenous HA-tagged MAPK or the mutants described in Fig 3C, in which either external lysines (MAPKKextR) or all lysines (MAPKnoK) were switched to arginines. MAPKnoK does not appear to be phosphorylatable by MEKEE. (C) Stably expressed MAPK mutants altering the proper folding of MAPK partially negate the impact of USP47 depletion. The L41E mutation is located in the kinase’s N-lobe, in the β1 strand preceding the glycine-rich ATP-phosphate-binding loop. The W225E is located in the αF helix that follows the activation segment and which forms the core of the C-lobe. Introduction of a charge residue (Glu) within these two key structural elements disrupts hydrophobic interactions and, hence, alters the structural integrity of the kinase domain. (D) Truncation of MAPK C-terminal tail also partially abrogates its sensitivity to USP47 depletion. Truncated MAPK was stably expressed in S2 cells and treated with the indicated dsRNAs. The Y329* truncation removes the C-terminal extension of MAPK, which includes helix αL16 that associates with the N-lobe. (E) The T and Y residues of MAPK’s activation segment are not required for regulation by Usp47. The TEY residues of MAPK’s activation segment at position 198–200 were replaced with AEF and stably expressed in S2 cells. Stably expressed HA-tagged wild-type MAPK is used as a control. (F) The ERK1/2 family D-site recruitment site that interacts with the docking motif of substrates and other ERK/MAPK interactors is not required for regulation by Usp47. The D-site recruitment site of Drosophila MAPK was inactivated by changing the D331 and D334 residues to asparagines. Exogenous MAPK modified in this manner was stably expressed [file pbio.1002539.s003.tif]

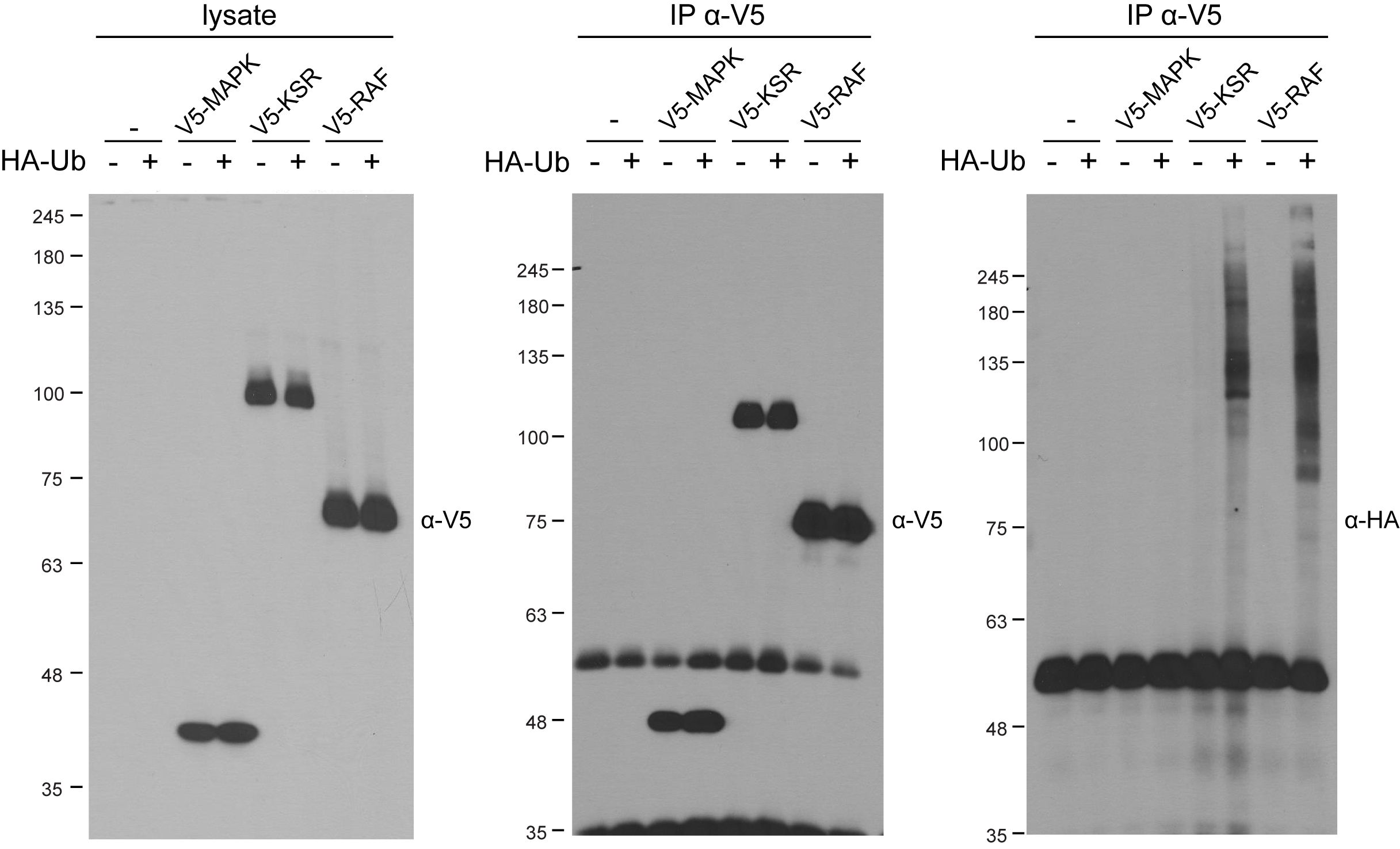

Supplement: S3 Fig — V5-tagged MAPK, KSR, and RAF were expressed alone or co-transfected with HA-tagged ubiquitin. Following α-V5 immunoprecipitation, samples were probed with α-HA to reveal polyubiquitinated forms (right panel). The α-V5 IP levels are shown in the middle panel, and lysates probed with α-V5 are shown in the left panel. (TIF) [file pbio.1002539.s004.tif]

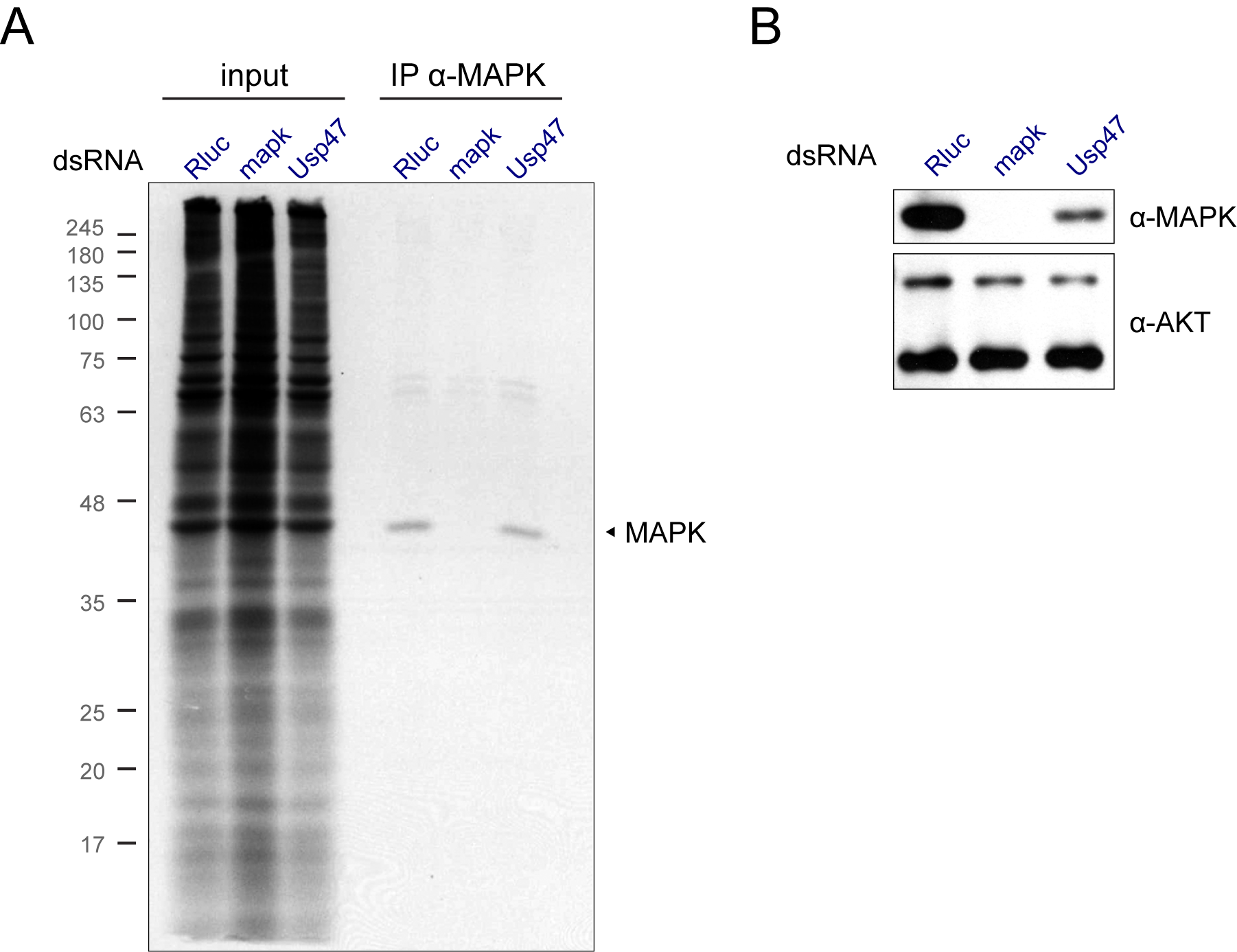

Supplement: S4 Fig — (A) S2 cells were treated with Rluc, mapk, or Usp47 dsRNA for 5 d, then were subjected to metabolic labeling with [35S]-methionine for 4 h. Radio-labeled (newly synthesized) MAPK was immunoprecipitated and detected by fluorography following western blotting. (B) Steady-state levels of MAPK from the samples shown in (A) were determined by western blotting to confirm their reduction upon USP47 depletion. Endogenous AKT is used as a loading control. (TIF) [file pbio.1002539.s005.tif]

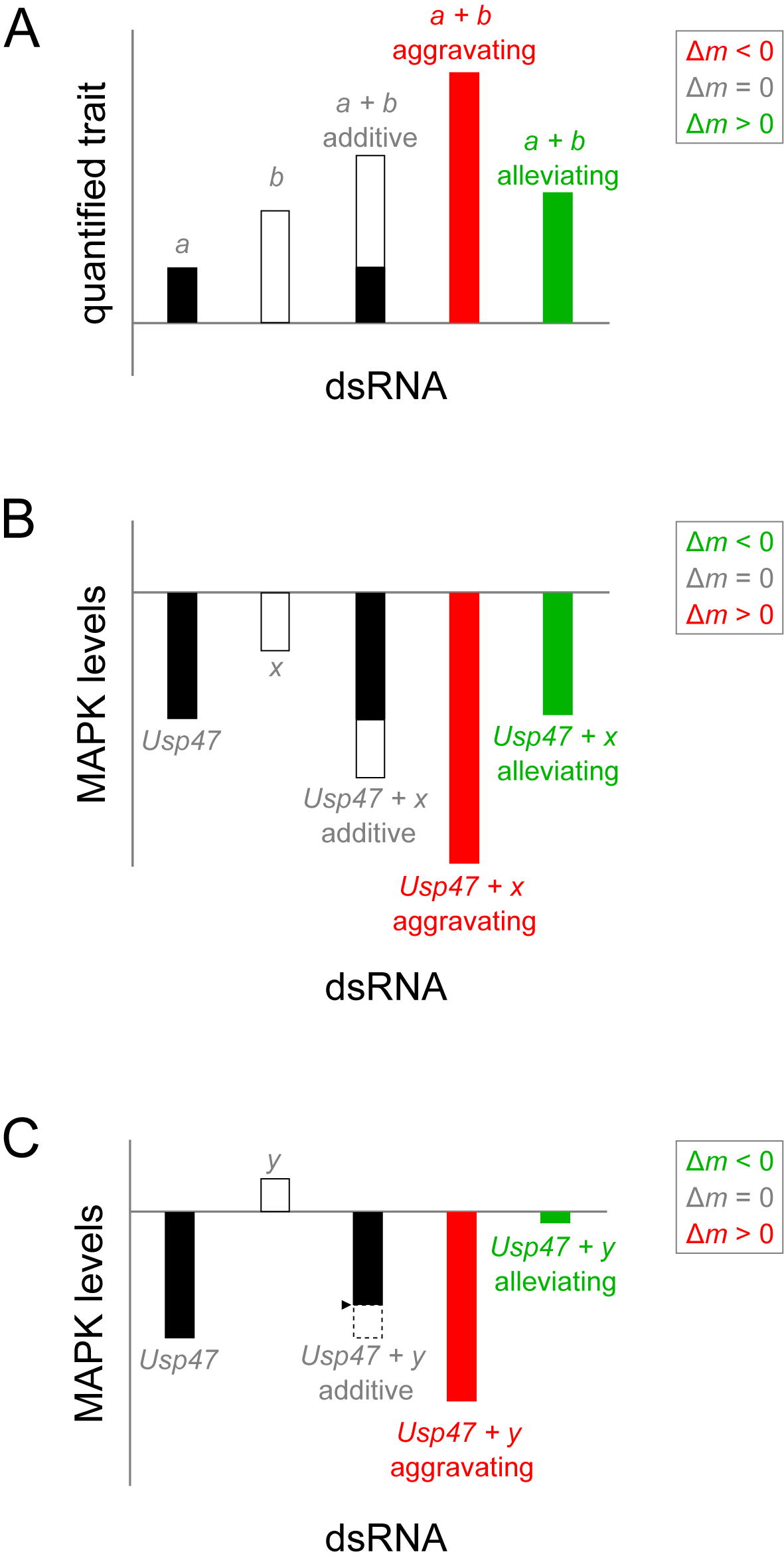

Supplement: S5 Fig — (A) Principle for defining a genetic interaction based on quantitative trait values obtained from single depletion of gene a and b as well as combined depletion. If the combined result is purely additive, there is no genetic interaction (Δm = 0). An aggravating genetic interaction takes place when the combined depletion value increases the severity of the phenotype beyond the expected additive value (this is also called positive epistasis; Δm < 0). Conversely, an alleviating effect takes place when the combined depletion is less severe than the expected additive value (negative epistasis; Δm > 0). The Δm is further explained in S1 Text. (B and C) Hypothetical genetic interaction examples in which a is replaced by Usp47 RNAi. Since Usp47 KD has a negative impact on MAPK levels, the Δm for alleviating and aggravating interactions will be of the opposite sign to those associated with gene a KD in (A). (B) In this example, an RNAi targeting gene x reduces MAPK levels. A purely additive result for Usp47 + x indicates that no genetic interaction is taking place and that gene x will slightly reduce MAPK levels irrespective of whether Usp47 is depleted or not. This might occur if a transcription or splicing factor that acts on mapk were to be co-depleted with Usp47; like Usp47, the factor also reduces MAPK levels, but functions in parallel, acting at a completely different regulatory step. However, if x aggravates the impact of Usp47 RNAi by decreasing MAPK levels beyond the expected additive value, then x interacts genetically with Usp47. An example of this would be a putative deubiquitinase acting redundantly with Usp47. On the contrary, an alleviating genetic interaction takes place if Usp47+x is lesser than that expected additive value. This might occur in the case of a factor acting in the same “pathway” as Usp47, for instance, a transcription factor promoting Usp47 expression or a factor stabilizing USP47 protein levels. (C) An RNAi targeting gene y slightly increases [file pbio.1002539.s006.tif]

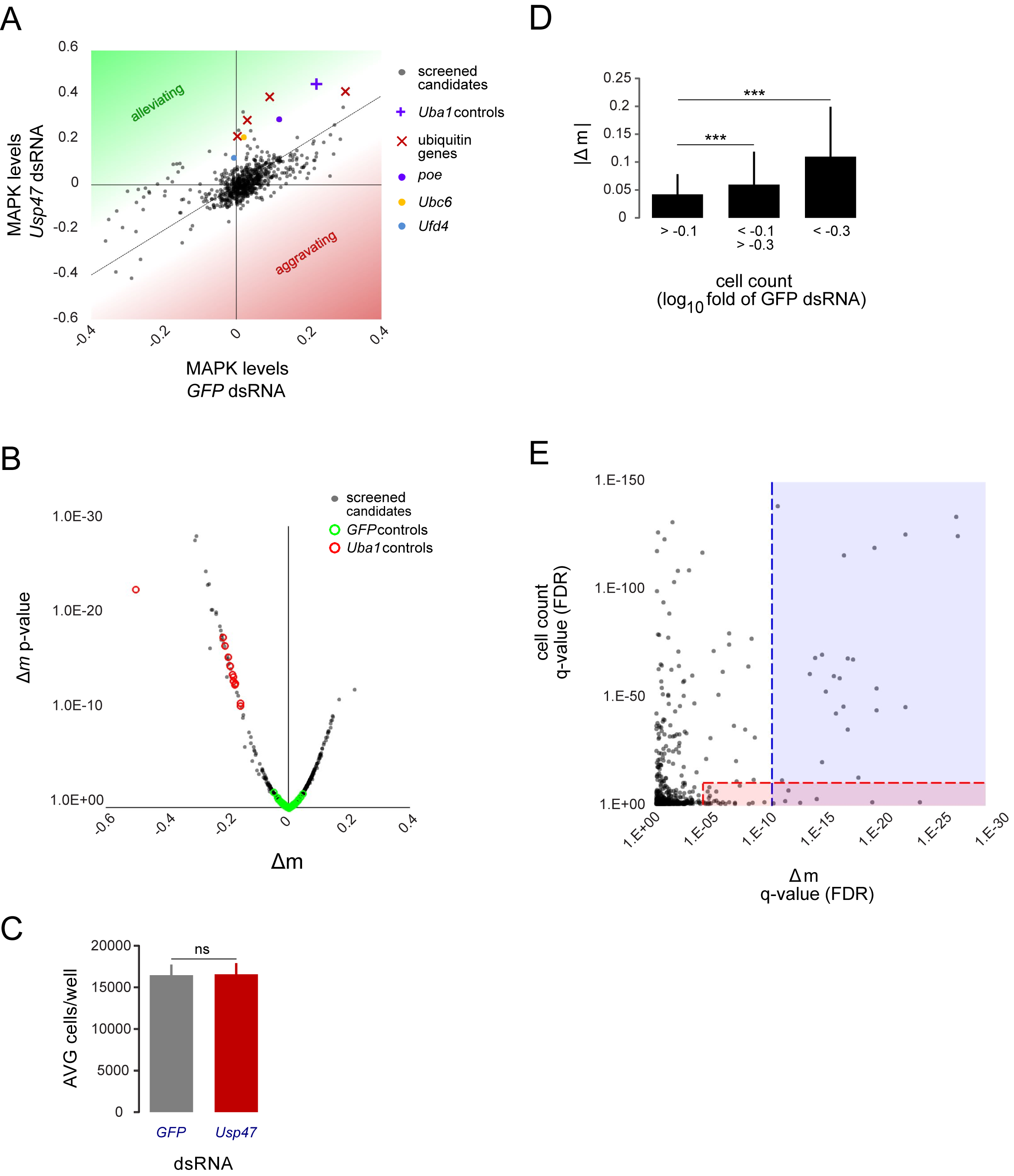

Supplement: S6 Fig — (A) MAPK levels from the Usp47 RNAi screen are plotted for GFP co-depletion samples against the corresponding Usp47 co-depletion samples. X-axis values are normalized to GFP dsRNA controls, while y-axis values are normalized to Usp47 dsRNA controls. In general, MAPK levels from Usp47 co-depletion samples generally mirror those of the GFP co-depletion controls (following the dashed diagonal). This suggests that most candidates do not differ from the expected neutral phenotype of a merely additive co-depletion effect. Genetic interactors of Usp47 are situated either above the diagonal (alleviating: green shaded area) or below the diagonal (aggravating: red shaded area). Ubiquitin genes, poe, Ubc6, and Ufd4, are highlighted as examples of factors that alleviate Usp47 RNAi. (B) Volcano plot of Δm scores plotted against Δm p-values. The two values are generally well correlated, prompting us to use the p-value to guide candidate selection. (C) Average cells per well imaged for both GFP and Usp47 co-depletion samples throughout the screen. The absence of a significant difference between the two suggests that there was not a widespread synthetic lethality effect caused by co-depletion of Usp47 and other ubiquitin-proteasome factors. (D) A reduction in cell count is correlated with a non-neutral Δm (***: p < 0.001). We therefore used cell count as a secondary criterion to increase the stringency of hit selection. (E) Graphical representation of the hit selection criteria. The false discovery rate (FDR) of Δm is plotted against the FDR of the cell count values. A global Δm cutoff (blue line) is used to select hits irrespective of their impact on viability (blue shaded area). A second less stringent Δm cutoff (red line) is used to select candidates that had little or no impact on cell count (red shaded area). Raw data for (B-D) can be found in S1 Data. Data related to (A and E) is contained in S1 Table. (TIF) [file pbio.1002539.s007.tif]

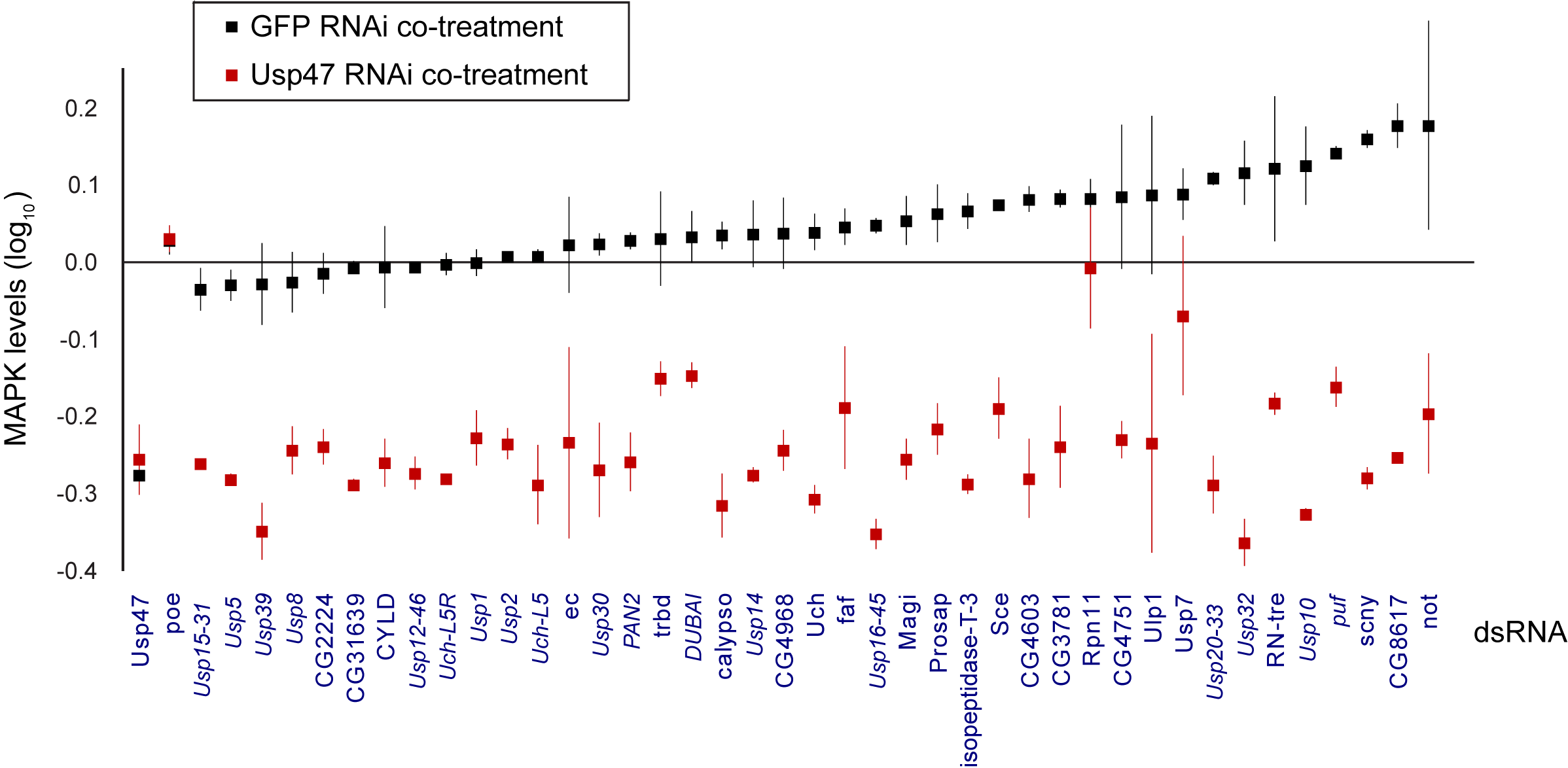

Supplement: S7 Fig — Results from the DUB co-depletion screen. Usp47 RNAi depletion has a partial impact on MAPK levels, even at prolonged depletion times. An independent set of dsRNAs targeting the predicted DUBs in Drosophila was designed to specifically re-screen these genes to look for any potential redundant factors that might act synergistically with Usp47. However, as was the case in the primary screen, no other DUB was found to cause an appreciable MAPK reduction, either on its own or in conjunction with Usp47 depletion. Rpn11 does, however, rescue MAPK levels in co-depletion with Usp47. This is most likely attributable to its function as a proteasome component. The data used to prepare this figure is contained in S3 Table. (TIF) [file pbio.1002539.s008.tif]

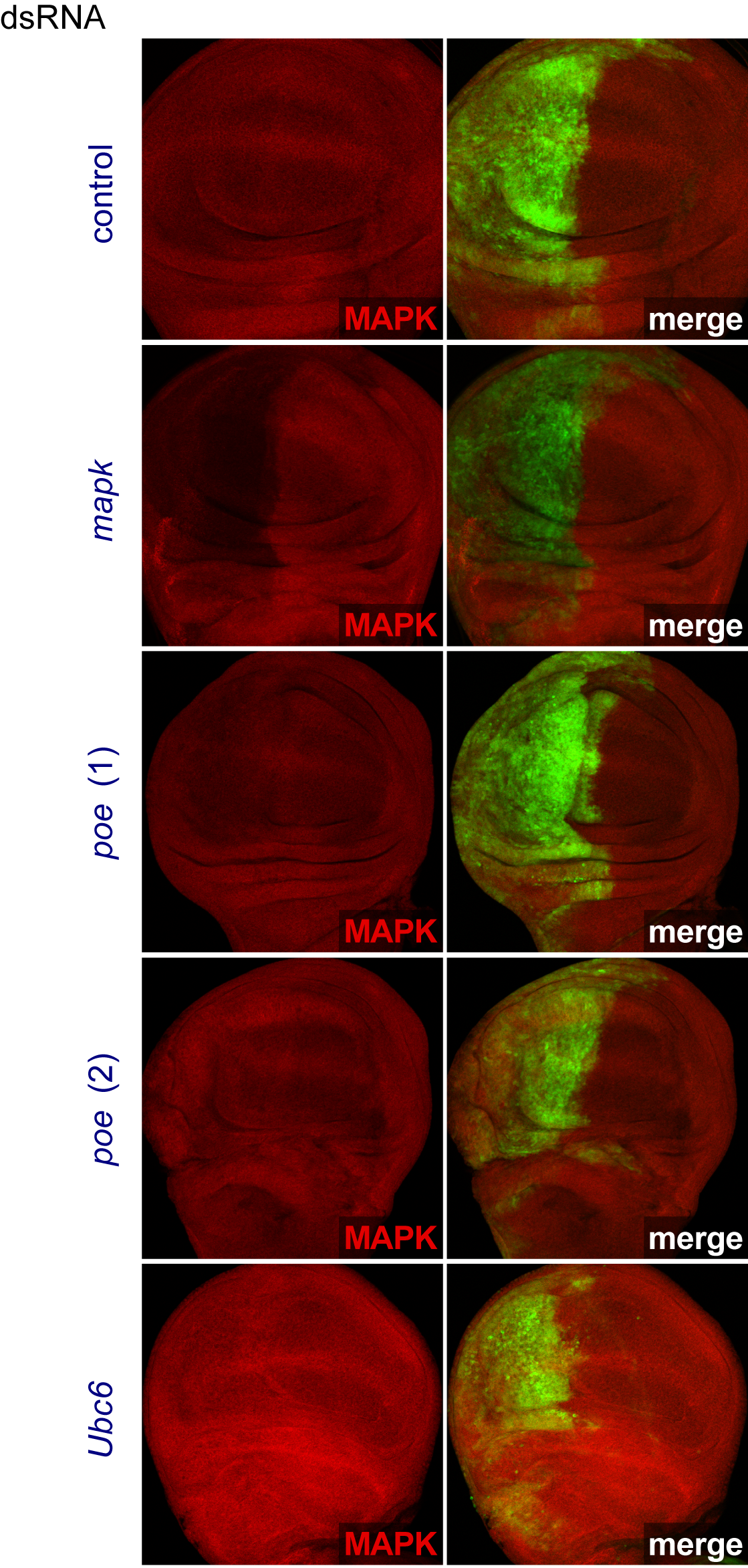

Supplement: S8 Fig — As a control for the dual RNAi depletions presented in Fig 5D, we depleted poe and Ubc6 alone, using the same RNAi lines. The engrailed-gal4 driver was used to drive expression of RNAi in the posterior segment (GFP-positive) of the disc. Ubc6 RNAi expression caused extensive larval lethality. Those wing discs that could be recovered were of reduced size. The RNAi lines used in this experiment correspond to the following VDRC lines: mapk, KK109108; poe (1), KK108296; poe (2), GD17648; Ubc6, GD23229. Canton-S flies were crossed to engrailed-gal4 flies as a negative control. (TIF) [file pbio.1002539.s009.tif]

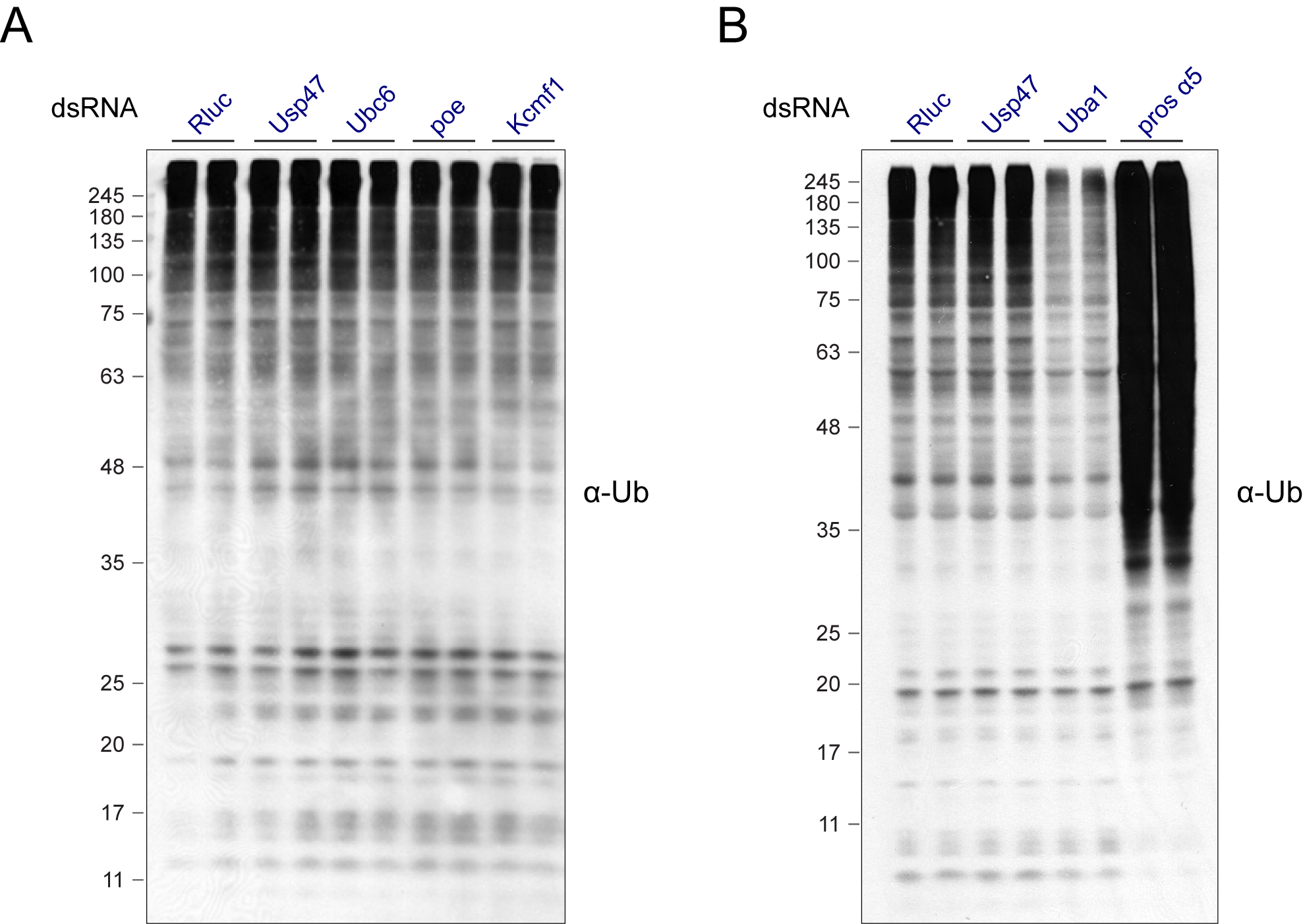

Supplement: S9 Fig — (A) S2 cells were treated with the indicated dsRNA and ubiquitin was subsequently examined by western blot (duplicate samples). USP47 and the E2/E3 ligases acting on MAPK did not visibly alter global protein ubiquitination. (B) Uba1 and pros α5 dsRNAs were used as positive controls for factors causing ubiquitin reduction and accumulation, respectively. (TIF) [file pbio.1002539.s010.tif]

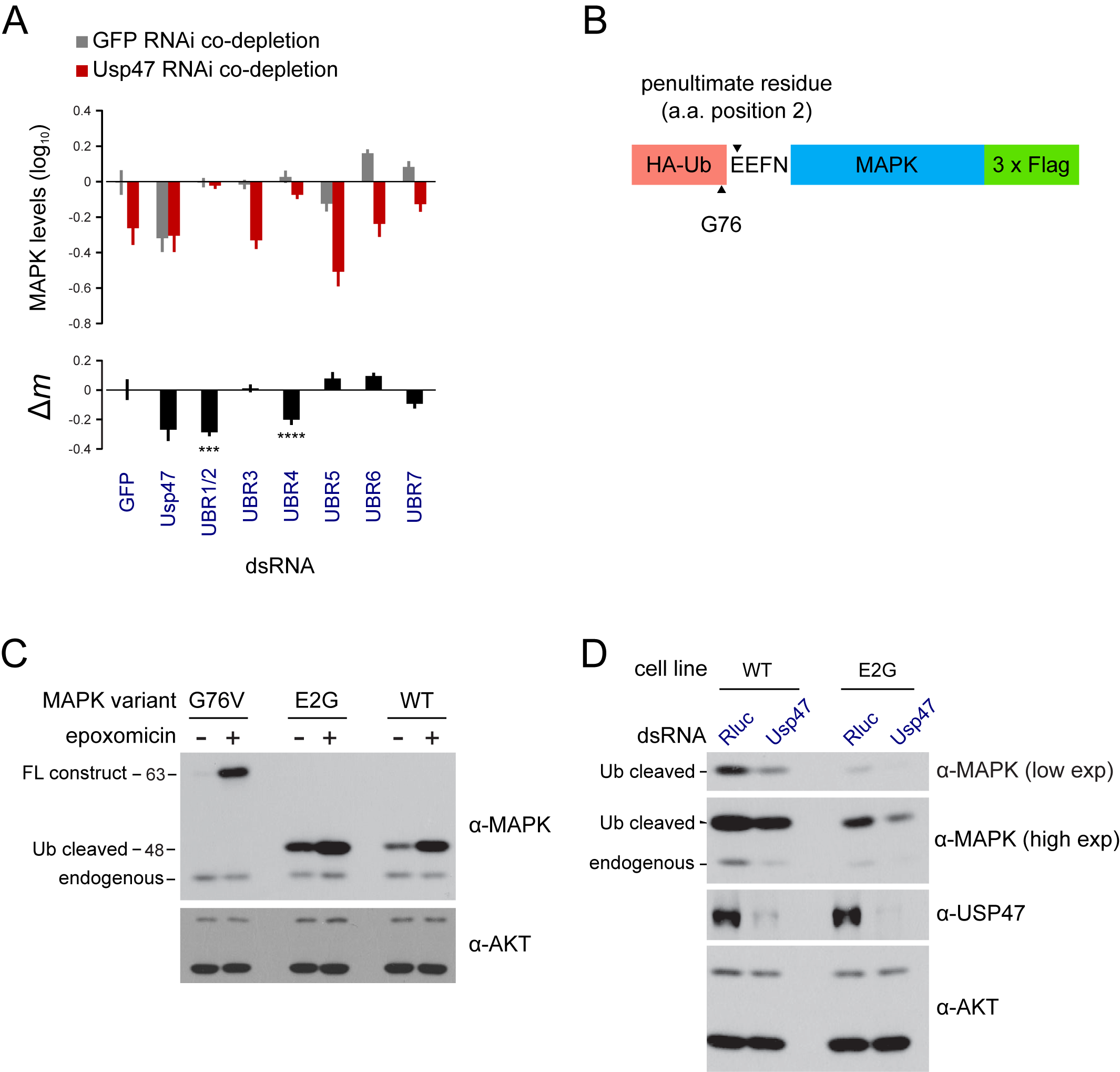

Supplement: S10 Fig — (A) We retested the Drosophila UBR family factors (N-recognins) to verify whether members of this family other than UBR4/POE also suppress the impact of Usp47 dsRNA on MAPK levels. Independent dsRNAs were designed for UBR1/2 (CG9086), UBR3 (CG42593), UBR4 (poe), UBR5 (hyd), UBR6 (FBX011), and UBR7 (CG15141). Besides UBR4/poe, only UBR1/2 dsRNA had a robust alleviating impact on MAPK levels when co-depleted with Usp47 (***: p < 0.001; ****: p < 0.0001). However, unlike UBR4, UBR1/2 RNAi caused a sharp reduction in cell count (see experimental data in S6 Table). (B) Diagram of the ubiquitin-fusion construct used to expose the glutamate residue following the initiator methionine (penultimate residue). Ubiquitin is cleaved by DUBs (following the G76 residue), exposing the penultimate residue. (C) The Ub-fusion MAPK protein is cleaved to expose the penultimate residue (the glutamate residue following the initiator methionine on WT MAPK). An uncleavable G76V Ub mutant is used as a control for co-translational Ub cleavage. The E2G MAPK mutant has the penultimate glutamate replaced with a glycine (a stabilizing residue). For this experiment, S2 cells were transiently transfected with the Ub-fusion constructs prior to treatment with epoxomicin or DMSO (controls). (D) Following cleavage of the N-terminal ubiquitin, both the WT (glutamate) and E2G mutant forms of MAPK are sensitive to Usp47 depletion. S2 cells were stably transfected with a Ub-fusion construct containing either the WT MAPK sequence (WT) or the E2G mutant form. These cell lines were then treated with Usp47 dsRNA for 4 d. Two exposures of the MAPK blot are presented as differences in expression levels of the Ub-MAPK fusions between both cell lines make it difficult to observe the Usp47 depletion effect on a single exposure. (TIF) [file pbio.1002539.s011.tif]
