# Supplementary material for: The Deubiquitinase USP47 Stabilizes MAPK by Counteracting the Function of the N-end Rule ligase POE/UBR4 in Drosophila
Source: PLoS Biol. 2016 Aug 23;14(8):e1002539. doi: 10.1371/journal.pbio.1002539 (PMC4994957; doi:10.1371/journal.pbio.1002539)
Supplement: S4 Table — (PDF) [file pbio.1002539.s015.pdf]

| gene | FBgn | # dsRNA probes confirming | reduced cell count | function |
|---|---|---|---|---|
| *CG31053* | FBgn0051053 | 1 | | encodes RING domain protein |
| *Ufd4* | FBgn0032208 | 1 | | UFD4/HECTD1 ortholog; HECT domain E3 ligase |
| *fzy* | FBgn0001086 | 1 | + | CDC20 homolog; member of the APC complex |
| *poe* | FBgn0011230 | 2 | | UBR4 ortholog; UBR box domain E3 ligase |
| *Prosalpha5* | FBgn0016697 | 2 | ++ | proteasome subunit |
| *Prosbeta1* | FBgn0010590 | 1 | ++ | proteasome subunit |
| *Prosbeta3* | FBgn0026380 | 2 | ++ | proteasome subunit |
| *Rpn2* | FBgn0028692 | 2 | ++ | proteasome regulatory subunit |
| *RpS27A* | FBgn0003942 | 2 | ++ | ubiquitin and ribosomal protein |
| *Ubc6* | FBgn0004436 | 1 | + | RAD6 ortholog; E2 ubiquitin ligase |
| *Ubi-p5E* | FBgn0086558 | 2 | ++ | ubiquitin |
| *Ubi-p63E* | FBgn0003943 | 2 | ++ | ubiquitin |
| *Ufd1-like* | FBgn0036136 | 2 | + | UFD1 ortholog; involved in endoplasmic reticulum associated degradation (ERAD) |
