# Supplementary material for: The Deubiquitinase USP47 Stabilizes MAPK by Counteracting the Function of the N-end Rule ligase POE/UBR4 in Drosophila
Source: PLoS Biol. 2016 Aug 23;14(8):e1002539. doi: 10.1371/journal.pbio.1002539 (PMC4994957; doi:10.1371/journal.pbio.1002539)
Supplement: S1 Text — (DOCX) [file pbio.1002539.s020.docx]

# Post Translational Control of MAPK Expression by the Deubiquitinase USP47 and N-end Rule Ubiquitin Ligases

## Supplemental material and methods

### Antibodies

Commercially available antibodies were used to detect the following *Drosophila* proteins: α-Ub (Santa Cruz Biotechnology, SC8017); α-MAPK (Cell Signaling, α-ERK1/2, #4695); α-MEK (Cell Signaling, #9122); α-AKT (Cell Signaling, #9272); α-ACTIN (EMD Millipore, #MAB1501); α-Alpha-TUBULIN (Sigma-Aldrich, #T9026). The α-HA, α-V5, α-CNK, α-RAS and α-RAF have been described previously [1]. α-USP47 (α-UBP64E) was obtained from C.P. Verrijzer [2]. Custom antibodies were generated against UFD4 (CG5604) and MAPK (for immunoprecipitation of endogenous MAPK) by Thermo Scientific using the following peptides: (UFD4: EEIMKERLLTATKEKGFHLN; MAPK: FEETLKFKERQPDNAP).

### Plasmids

Plasmids used in transfection experiments and to generate stable cell lines were derived from a *pAct5C* vector, excepting for the V5 epitope-tagged *Raf*, *ksr* and *mapk* as well as *mek^EE^* which were cloned into a copper-inducible *pMet* vector that has been previously described [1]_._ The *poe*, *kcmf1* and *HA-mapk* constructs were prepared and inserted into the *pAct5C* vector according to standard molecular biology procedures. Protein expression using the *pMet* vector was induced by adding CuSO_4_ (0.7 mm) to the medium either 24h prior to cell lysis (for the V5-tagged constructs) or 36h prior to lysis (*mek^EE^*). *M. musculus* ERK1 and human ERK2 vectors were obtained from S. Meloche (Université de Montréal) and transferred into the *Drosophila* *pAct5C* vector in order to generate stable cell lines. Ub-fusion constructs (*HA-Ub*-*mapk-3xFLAG*) were generated using human HA-tagged ubiquitin which was fused to fly *mapk* (in the *pAct5C* vector) according to the specifications in [3] to allow for co-translational cleavage of the N-terminal Ub. Point mutations and deletions were generated using QuickChange (Stratagene). The position of changed residues and stop codon insertions are indicated in the figures and figure legends.

### Targeted RNAi Library

Candidate selection for factors associated to UPS function was performed using Flymine [4] to select for specific GO terms as well as the presence of annotated domains associated to E2 and E3 activity. Additionally, the orthologs of human and *S. cerevisiae* genes with GO terms associated to ubiquitination were also included. Finally, genes from the DRSC [5] targeted screening set for ubiquitin were also included in our list. The targeted dsRNA library was generated from dsDNA templates originally purchased from Open Biosystems (Huntsville, AL; http://www.openbiosystems.com) and is described in detail in [6]. In-house dsRNA reagents were also added to supplement the collection. Follow-up dsRNAs were designed with the help of E-RNAi software [7] . A detailed list of primer sequences for the dsRNAs used in this study is provided in Table S7.

### Genetic Interaction Score

For the purposes of the RNAi screen, genetic interaction was defined as deviation from the expected result (neutral phenotype), which is a common definition of genetic interaction when working with quantitative phenotypes (MAPK levels in our case) [8]. The expected result is obtained by adding the single RNAi depletion effects together. The combined depletion effect can then be compared to the expected result. The genetic interaction score, Δ*m*, is thus derived as follows:

$$\Delta m=\log\left( \frac{m_{x}}{m_{g}} \right)-\log\left( \frac{m_{xu}}{m_{gu}} \right)$$

Where *m_x_* is the measured MAPK signal upon knocking down a given gene *x* and *m_g_* is the MAPK signal for *GFP* RNAi controls. *m_xu_* is the MAPK signal for co-depletion of gene x with *Usp47*. *m_gu_* is the MAPK signal of *GFP* and *Usp47* co-depletion controls. Thus, a dsRNA whose impact on MAPK levels is purely additive with *Usp47* dsRNA will have a Δ*m* = 0 (no genetic interaction). Conversely, non-additive co-depletion effects will produce Δ*m* <> 0 and are indicative of genetic interaction with *Usp47*.

A negative Δ*m* would be obtained in the case of factors that alleviate *Usp47*’s effect on MAPK (alleviating genetic interaction). These would include factors, such as *Uba1*, that restore MAPK levels by partially or completely negating *Usp47*’s impact (alleviating rescue effect; the factor is epistatic to *Usp47*) and also potential redundant factors, such as DUB, whose co-depletion with *Usp47* would be less than the sum of the individual RNAi (alleviating redundant).

On the other hand, a positive Δ*m* would occur in cases where the impact on MAPK levels in *Usp47* co-depletion is synergistic or greater than the sum of the individual depletion effects (synthetic genetic interaction). For instance, a potential factor acting redundantly with *Usp47* to stabilize MAPK might have little impact when depleted on its own. However, in the absence of *Usp47*, depleting this factor might now cause a further drop in MAPK levels (synthetic redundant). Another case would be a negative regulator of *Usp47* whose impact on MAPK levels is negated by *Usp47* co-depletion (*Usp47* would be epistatic to this factor in this case).

### Candidate Selection Criteria

For the *Usp47* co-depletion screen, we used a dual cutoff based hit selection strategy. Hits with a Δ*m* false discovery rate (FDR) below 1x10^-10^ were retained irrespective of other parameters. Hits with a Δ*m* FDR between 1x10^-10^ and 1x10^-3^ were only retained if their cell count FDR was also above 1x10^-10^ (Fig S6E). This second cutoff critera was introduced to bias hit selection towards factors that did not cause a significant change in cell count but had a weaker confidence Δ*m*.

An RNAi validation experiment was then conducted on selected hits. For this, we synthesized two separate dsRNA reagents per candidate that differed from the primary screen dsRNA and, when possible, did not overlap with its sequence. The confirmation criteria used for validation were a Δ*m* P value < 0.05 and an absolute change in MAPK levels above 0.1 (log_10_ normalized). Validated hits also were required to have an identical type of genetic interact (aggravating vs. alleviating) to that of the primary screen (same sign Δ*m*). A number of proteasome components failed on this last criteria, possibly due to their broader impact that includes cell lethality; it is conceivable that an alleviating impact may be observed following depletion followed by a drop in MAPK levels due to the general impact of cell death on protein stability due to caspases, for example. Notably, none of the positive Δ*m* candidates (synergistic interactions) were confirmed in our validation experiments. Subsequent to the validation step, follow-up experiments were conducted using one of the two dsRNAs used for validation.

## SUPPLEMENTARY REFERENCES

1. Douziech M, Roy F, Laberge G, Lefrancois M, Armengod AV, Therrien M. Bimodal regulation of RAF by CNK in Drosophila. Embo J. 2003;22(19):5068-78. PubMed PMID: 14517245.

2. Bajpe PK, van der Knaap JA, Demmers JA, Bezstarosti K, Bassett A, van Beusekom HM, et al. Deubiquitylating enzyme UBP64 controls cell fate through stabilization of the transcriptional repressor tramtrack. Mol Cell Biol. 2008;28(5):1606-15. Epub 2007/12/28. doi: 10.1128/MCB.01567-07. PubMed PMID: 18160715.

3. Varshavsky A. Ubiquitin fusion technique and related methods. Methods Enzymol. 2005;399:777-99. doi: 10.1016/S0076-6879(05)99051-4. PubMed PMID: 16338395.

4. Lyne R, Smith R, Rutherford K, Wakeling M, Varley A, Guillier F, et al. FlyMine: an integrated database for Drosophila and Anopheles genomics. Genome Biol. 2007;8(7):R129. Epub 2007/07/07. doi: gb-2007-8-7-r129 [pii] 10.1186/gb-2007-8-7-r129. PubMed PMID: 17615057.

5. Flockhart IT, Booker M, Hu Y, McElvany B, Gilly Q, Mathey-Prevot B, et al. FlyRNAi.org--the database of the Drosophila RNAi screening center: 2012 update. Nucleic Acids Res. 2012;40(Database issue):D715-9. doi: 10.1093/nar/gkr953. PubMed PMID: 22067456; PubMed Central PMCID: PMC3245182.

6. Goshima G, Wollman R, Goodwin SS, Zhang N, Scholey JM, Vale RD, et al. Genes required for mitotic spindle assembly in Drosophila S2 cells. Science. 2007;316(5823):417-21. Epub 2007/04/07. doi: 1141314 [pii] 10.1126/science.1141314. PubMed PMID: 17412918.

7. Horn T, Boutros M. E-RNAi: a web application for the multi-species design of RNAi reagents--2010 update. Nucleic Acids Res. 2010;38(Web Server issue):W332-9. Epub 2010/05/07. doi: gkq317 [pii] 10.1093/nar/gkq317. PubMed PMID: 20444868; PubMed Central PMCID: PMC2896145.

8. Mani R, St Onge RP, Hartman JLt, Giaever G, Roth FP. Defining genetic interaction. Proc Natl Acad Sci U S A. 2008;105(9):3461-6. Epub 2008/02/29. doi: 10.1073/pnas.0712255105. PubMed PMID: 18305163; PubMed Central PMCID: PMC2265146.
